# Kinesin and myosin motors compete to drive rich multiphase dynamics in programmable cytoskeletal composites

**DOI:** 10.1093/pnasnexus/pgad245

**Published:** 2023-07-31

**Authors:** Ryan J McGorty, Christopher J Currie, Jonathan Michel, Mehrzad Sasanpour, Christopher Gunter, K Alice Lindsay, Michael J Rust, Parag Katira, Moumita Das, Jennifer L Ross, Rae M Robertson-Anderson

**Affiliations:** Department of Physics and Biophysics, University of San Diego, San Diego, CA 92110, USA; Department of Physics and Biophysics, University of San Diego, San Diego, CA 92110, USA; School of Physics and Astronomy, Rochester Institute of Technology, Rochester, NY 14623, USA; Department of Physics and Biophysics, University of San Diego, San Diego, CA 92110, USA; Department of Mechanical Engineering, San Diego State University, San Diego, CA 92182, USA; Department of Physics, Syracuse University, Syracuse, NY 13244, USA; Department of Molecular Genetics and Cell Biology, University of Chicago, Chicago, IL 60637, USA; Department of Mechanical Engineering, San Diego State University, San Diego, CA 92182, USA; School of Physics and Astronomy, Rochester Institute of Technology, Rochester, NY 14623, USA; Department of Physics, Syracuse University, Syracuse, NY 13244, USA; Department of Physics and Biophysics, University of San Diego, San Diego, CA 92110, USA

**Keywords:** cytoskeleton, active matter, kinesin, actin, microtubules

## Abstract

The cellular cytoskeleton relies on diverse populations of motors, filaments, and binding proteins acting in concert to enable nonequilibrium processes ranging from mitosis to chemotaxis. The cytoskeleton's versatile reconfigurability, programmed by interactions between its constituents, makes it a foundational active matter platform. However, current active matter endeavors are limited largely to single force-generating components acting on a single substrate—far from the composite cytoskeleton in cells. Here, we engineer actin–microtubule (MT) composites, driven by kinesin and myosin motors and tuned by crosslinkers, to ballistically restructure and flow with speeds that span three orders of magnitude depending on the composite formulation and time relative to the onset of motor activity. Differential dynamic microscopy analyses reveal that kinesin and myosin compete to delay the onset of acceleration and suppress discrete restructuring events, while passive crosslinking of either actin or MTs has an opposite effect. Our minimal advection–diffusion model and spatial correlation analyses correlate these dynamics to structure, with motor antagonism suppressing reconfiguration and demixing, while crosslinking enhances clustering. Despite the rich formulation space and emergent formulation-dependent structures, the nonequilibrium dynamics across all composites and timescales can be organized into three classes—slow isotropic reorientation, fast directional flow, and multimode restructuring. Moreover, our mathematical model demonstrates that diverse structural motifs can arise simply from the interplay between motor-driven advection and frictional drag. These general features of our platform facilitate applicability to other active matter systems and shed light on diverse ways that cytoskeletal components can cooperate or compete to enable wide-ranging cellular processes.

**Significance Statement**

The cytoskeleton is a paradigmatic active matter system—comprising protein filaments, motors, and crosslinkers—that mediates wide-ranging cellular processes from migration to morphogenesis. The cytoskeleton's composite nature, conferring versatility and programmability, is one of its hallmarks. Yet, state-of-the-art active matter designs are largely limited to single force-generating components and substrates. Here, we engineer composites of MTs and actin driven by kinesin and myosin motors to restructure, contract, and flow to form structures ranging from interpenetrating scaffolds to phase-separated clusters. Surprisingly, kinesin and myosin compete to delay rapid restructuring and suppress demixing. Our bioinspired nonequilibrium composites not only bring reconstituted systems a critical step closer to mimicking cytoskeletal complexity but are also foundational for diverse material applications from wound healing to soft robotics.

## Introduction

The cytoskeleton is a dynamic, nonequilibrium material comprising protein filaments, including actin, microtubules (MTs), and intermediate filaments, as well as motor proteins, such as myosins and kinesins, that actively push and pull on the protein filaments (1–8). Crosslinking proteins also connect and bundle filaments as needed for cellular processes (9–12). This complex composite continuously restructures and reconfigures in response to demands of the cell to enable diverse processes from cytokinesis to mechanosensing (3–5, 7, 8, 13–21). In vitro systems of reconstituted cytoskeletal proteins, which display rich and tunable dynamics, are also intensely studied as model active matter platforms to shed light on the nonequilibrium physics underlying force-generating, reconfigurable systems (7, 12, 19, 22–40).

Interacting networks of semiflexible actin filaments and rigid MTs provide tensile and compressive strength to the cytoskeleton while allowing for cell mobility, key to processes such as division and chemotaxis (15, 16, 41–45). Further, recent studies have shown that in vitro actin–MT composites exhibit emergent mechanical properties that are not a simple sum of the single component systems (46–48). For example, composites with comparable concentrations of actin and MTs display both enhanced filament mobility and increased stiffness (46), as well as an emergent nonmonotonic dependence of elasticity on actin crosslinking (47).

More recently, myosin II minifilaments have been incorporated into actin–MT composites, showing that synergistic interactions between actin and MTs prevent disordered flow and rupturing often seen in actomyosin networks without crosslinkers (26–28). These studies have also shown that composites can be tuned to display enhanced mechanical strength (27), coordinated motion of actin and MTs, sustained ballistic contraction, and mesoscale restructuring (26, 28)—all in the absence of crosslinking proteins to chemically connect the filaments.

Microtubule-based active matter systems have also been engineered using clusters of kinesin motors that crosslink and pull on microtubule bundles to create active nematics (23, 24, 30, 31, 34, 35, 49–55). In these systems, kinesins generate long-lasting turbulent flows by cyclically extending, buckling, fracturing, and healing bundles (49). More recently, actin has been incorporated into active microtubule fluids, resulting in turbulent flow, contraction, or formation of layered asters (29).

The distinct dynamics and structures that kinesin-driven and myosin-driven systems display beg the question as to how different active components and substrates cooperate or compete to control cellular processes. While composite active matter is beginning to be developed to introduce more control and tunability over single-substrate systems (26–29, 56), incorporating two active components that act on distinct substrates represents a paradigm shift in active matter. Beyond the cellular relevance, such designs can elucidate general principles for nonequilibrium programmable materials that can reconfigure and generate force and determine how to enhance programmability and expand the dynamical and structural phase space by altering the active and static nature of crosslinkers and the substrates on which they act.

Here, we engineer actin–MT composites that undergo a rich combination of advective flow, contraction, and multimode restructuring driven by kinesin *and* myosin motors. These dynamics are coupled to distinct time-evolving structures that range from interpenetrating actin–MT scaffolds to microscale phase-separated amorphous clusters. We couple differential dynamic microscopy (DDM) with particle image velocimetry (PIV) to discover that competition between kinesin–MT activity and actomyosin activity delays the onset of rapid restructuring while crosslinking of either actin or MTs accelerates the time evolution of active dynamics. Our advection–diffusion model and spatial correlation analyses correlate the dynamic antagonism that we observe with suppressed demixing of double-motor composites and the crosslinker-mediated acceleration with enhanced restructuring and clustering. Despite these complexities, we find that the broad phase space of active dynamics can be organized into three general classes with distinct types and rates of ballistic motion.

## Results and discussion

### Active cytoskeleton composite design and formulation–structure phase space

**Fig. 1.**
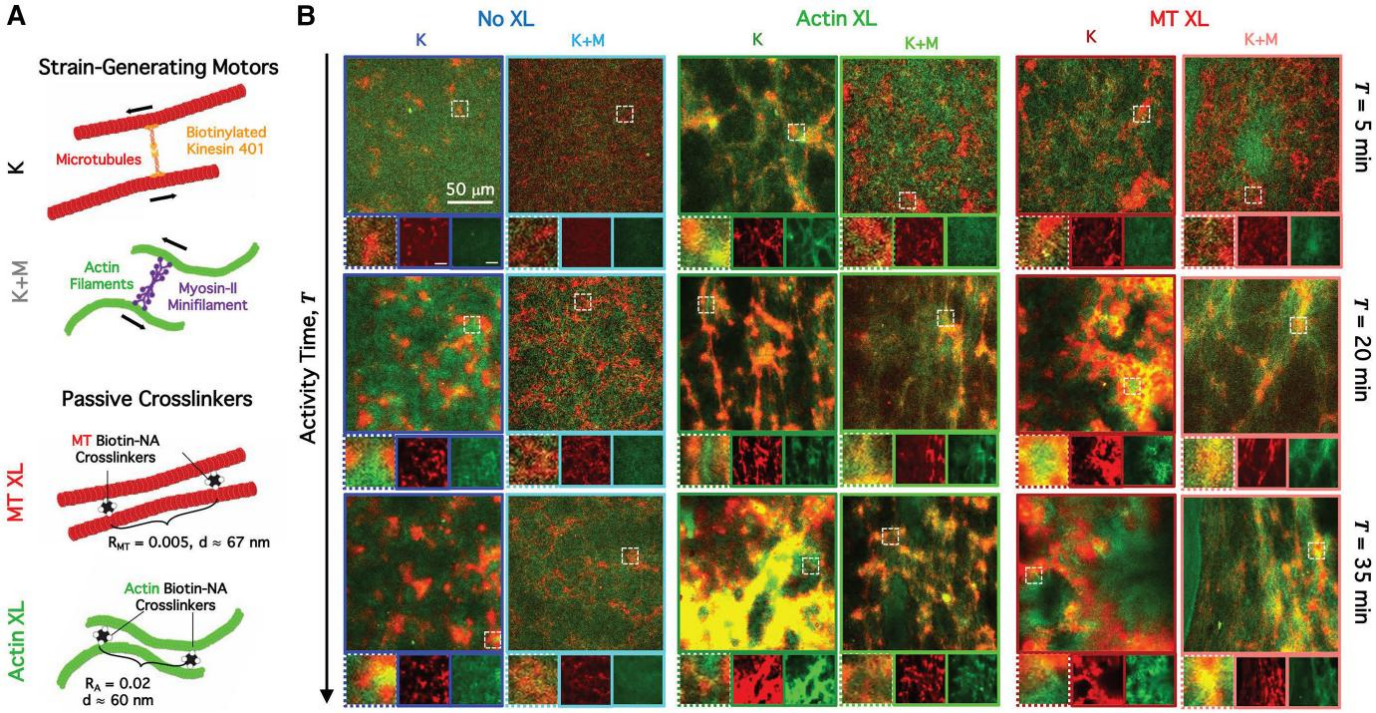
Engineering and characterizing active cytoskeleton composites with varying strain-generating components and connectivity. (A) We co-polymerize actin monomers (2.32 µM) with tubulin dimers (3.48 µM) to form co-entangled composite networks of actin filaments and MTs. We use NeutrAvidin to passively crosslink biotinylated actin filaments (Actin XL) or MTs (MT XL) at crosslinker:protein molar ratios of $R_{A}$ = 0.02 and $R_{\mathrm{MT}}$ = 0.005 for actin and MTs to achieve similar distances *d* between crosslinks (48). We incorporate kinesin clusters and myosin II minifilaments to drive composites out of steady-state. (B) We acquire two-color confocal time-series of actin (green) and MTs (red) to capture motor-driven dynamics and reconfiguration. Each column includes images taken at three different time points, *T* = 5, 20, and 35 min, during motor activity for composites with kinesin (K, columns 1, 3, and 5), kinesin and myosin (K + M, columns 2, 4, and 6), no crosslinking (No XL, columns 1 and 2), actin crosslinking (Actin XL, columns 3 and 4), and MT crosslinking (MT XL, columns 5 and 6). Below each composite image is a zoom-in of a 25 µm × 25 µm region denoted by a dashed-line box in the main image and single-channel images showing separately the MTs (middle, red) and actin (right, green). The 50-µm scale bar in the top right panels applies to all full-size images.

We first describe our design strategy for realizing an active matter system that has two force-generating components that act on two distinct, yet homogenously co-mixed, substrates. Namely, we engineer composites of co-entangled MTs and actin (46) and incorporate kinesin clusters and myosin II minifilaments that crosslink and push and pull on pairs of MTs and actin, respectively, to generate force and motion (49, 57) (Fig. 1A; Fig. S1). To investigate the extent to which actomyosin and kinesin–MT activities act synergistically or antagonistically to dictate dynamics, we perform experiments with either kinesin (K, Fig. 1B) or both kinesin and myosin (K + M, Fig. 1B). We further characterize the effect of passive crosslinking of the MTs (MT XL, Fig. 1) or actin (Actin XL, Fig. 1) at crosslinker:protein molar ratios R that are high enough to induce measurable changes in the viscoelastic properties but low enough to prevent filament bundling (47). To observe the dynamics of our active cytoskeleton composites, we collect sequential two-color time-series of actin and MTs comprising composites over the ∼1-h time course of measurable active dynamics. As shown in Fig. 1B, by simply incorporating or omitting myosin motors and passive crosslinkers, we are able to drive substantial changes in the active restructuring, emergent dynamics, and programmable phase space of non-equilibrium properties (Movies S1–S3).

All composites begin in similar structural states with interpenetrating networks of actin and MTs uniformly distributed throughout the field of view (Fig. 1B, top row). However, each composite formulation reconfigures into distinct structural states over activity times of T≈30 min, where T=0 is defined as the time at which kinesin is added to the composite. While we do not visualize the motors, the spatially uniform active dynamics that we see at the onset of activity indicate that, like the filaments, the motors are uniformly mixed throughout the composite.

Examining the three kinesin-only composites (no myosin), we find that without passive crosslinkers, composites form loosely connected MT-rich amorphous clusters. Actin filaments first co-localize in the cluster centers but are then squeezed out into the surrounding space as the clusters contract further and disconnect from one another (Fig. 1B, dark blue boxes). Passive actin crosslinking hinders this separation of actin and MTs, enabling the slow uptake of actin into MT-rich clusters, such that the composite becomes a connected network of clusters of co-localized actin and MTs (Fig. 1B, dark green boxes). MT crosslinking leads to similar amorphous MT clustering and actin–MT de-mixing as without crosslinking; but these MT-rich regions coalesce over time, resulting in larger-scale phase separation of actin and MTs compared to the non-crosslinked case (Fig. 1B, dark red boxes).

Turning to the double-motor composites that incorporate both kinesin and myosin, we find that the addition of myosin impedes the kinesin-driven de-mixing described above and reduces the degree of restructuring over the course of activity (Fig. 1B, light shaded boxes). This effect can be seen in the images at all time points (rows in Fig. 1B), in which actin and MT networks are more evenly distributed and interpenetrating than composites without myosin. Without passive crosslinkers, composites show little rearrangement (Fig. 1B, light blue boxes), as seen in previous experiments on myosin-driven actin–MT composites (26–28). Crosslinking of actin or MTs enables more restructuring of the double-motor composites, but this reconfiguration and demixing is still more subdued than that for kinesin-only composites (Fig. 1B, light green and red boxes).

### Actin and MTs exhibit three distinct classes of coordinated ballistic dynamics

**Fig. 2.**
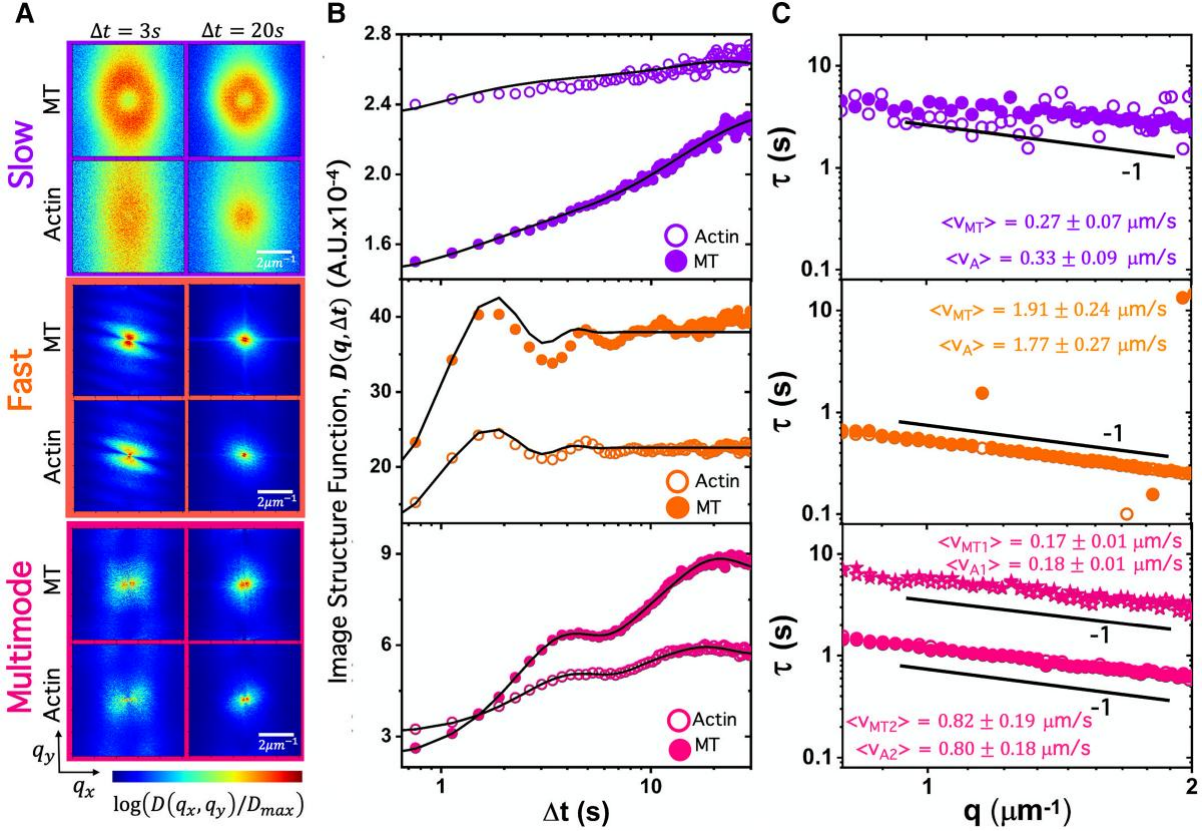
DDM reveals ballistic dynamics of composites that separate into three dynamically distinct classes. (A) Representative two-dimensional (2D) image structure functions $D(q_{x},q_{y})$ computed from the ensemble average of all Δt = 3 s (left) and Δt = 20 s (right) lag times for three representative videos (see Movies S1–S3). The color scale is normalized separately for each image and indicates the value of each image structure function $D(q_{x},q_{y},\Delta t)/D_{\max}$, with blue/red values indicating low/high correlations. (B) Azimuthally averaged 1D image structure functions D(q,Δt) versus lag time Δt computed from 2D $D(q_{x},q_{y},\Delta t)$ functions for MTs (closed symbols) and actin (open symbols) at wave vector $q=1.33\;\mu m^{-1}$. Black lines are fits to functions with Schulz speed distributions. (C) Corresponding decay times τ(q) computed from D(q,Δt) fits universally follow $\tau (q)=(vq{)}^{-1}$ scaling, indicative of ballistic motion. Speeds for actin ($v_{A}$) and MTs ($v_{\mathrm{MT}}$) determined from each τ(q) fit are listed. Listed error values are the standard deviation of the corresponding Schulz speed distribution.

To determine the non-equilibrium dynamics that enable this rich formulation-dependent restructuring, i.e. how the system gets from one structural state to another, we perform DDM on the actin and MT channels of each two-channel (i.e. two-color) video. As we describe in Methods and Supplementary material, DDM analyzes differences of images separated by varying lag times Δt in Fourier space to compute image structure functions $D(\vec{q},\Delta t)$ for different wave vectors $\vec{q}$, which describe how density fluctuations become decorrelated for a given spatial scale (i.e. 2π/q) (Fig. 2A and B).

Figure 2A shows two-dimensional (2D) image structure functions $D(q_{x},q_{y},\Delta t)$ computed for the MT and actin channels of three videos that are representative of different types of dynamics we measure, which we describe below. The plots in the left and right columns correspond to $D(q_{x},q_{y})$ for sample “short” and “long” lag times, Δt=3s and Δt=20s, and the color is set by the value of $D(q_{x},q_{y})$, with low (blue) and high (red) values indicating lower and higher correlations, respectively (see Fig. S2 for more $D(q_{x},q_{y})$ examples). The first notable feature in Fig. 2A (and Fig. S2) is the similarity in the functional form of $D(q_{x},q_{y})$ for actin and MT channels of the same video and lag time, indicating that the actin and MT network dynamics are well coupled despite cases in which we observe large-scale de-mixing (Fig. 1B). The lower magnitudes of D for actin compared to MTs are due to the comparatively lower signal of the actin channel. Moreover, the more uniform $D(q_{x},q_{y})$ values seen in the purple-bordered plots labeled “*Slow*,” as compared to the middle (orange, *Fast*) and bottom (magenta, *Multimode*), are indicative of more homogeneous and slow motion, in which fluctuations decorrelate less over a given lag time and over varying lengthscales (i.e. wave vectors). The modest radial asymmetry seen most clearly in the orange-bordered plots is a sign of anisotropic motion, which we discuss in later sections. Finally, the reduced correlation at Δt=20s compared to Δt=3s indicates that the decorrelation timescales are <20s.

To quantify the dynamics represented in Fig. 2A, we azimuthally average each $D(q_{x},q_{y},\Delta t)$ to compute a corresponding one-dimensional (1D) function for each lag time, D(q,Δt). Figure 2B shows sample D(q,Δt) curves for the three videos analyzed in Fig. 2A. We use the distinct functional features of these curves to organize our data for all composite formulations and activity times into three classes: *Slow* (top), *Fast* (middle), and *Multimode* (bottom). *Slow* curves show a simple slow rise to plateau at large lag times (Fig. 2B, top panel); *Fast* curves exhibit oscillations in the decorrelation plateau (Fig. 2B, middle panel); and *Multimode* curves reveal two distinct plateaus at well-separated lag times (Fig. 2B, bottom panel).

These nontrivial functional forms cannot be accurately described by exponential functions typically used in DDM (26, 28, 58, 59), so we instead use a function that assumes Schulz distributions of speeds, as has been used in other ballistic biological systems such as swimming *Escherichia coli* (60, 61) (see Methods and Supplementary material). This function captures the oscillatory plateaus seen in the *Fast* class, and a sum of two Schulz speed distributions accurately captures the two-plateau behavior of the *Multimode* class.

From the D(q,Δt) fits, we extract decay times, τ(q), which exhibit a power-law dependence on *q* that further quantifies the type and rate of motion (Fig. 2C). Despite the varied functional forms of the D(q,Δt) curves shown in Fig. 2B, the corresponding τ(q) for each class exhibits power-law scaling of $\tau (q)\;∼\;q^{-1}$, indicative of ballistic motion for both actin and MTs. Similar ballistic-like dynamics have been previously reported for myosin-driven cytoskeleton composites (26, 28). Fitting each τ(q) curve to the power-law relation $\tau (q)≃{\left(\langle v\rangle q\right)}^{-1}$ yields the average speed ⟨v⟩ of each filament type measured over the course of the corresponding video. As listed in Fig. 2C, we find that ⟨v⟩ for the *Fast* class is ∼7× larger than the *Slow*⟨v⟩. Fitting the *Multimode*D(q,Δt) data results in two distinct τ(q) curves with corresponding ⟨v⟩ values that differ ≳4-fold, suggesting that *Multimode* composites undergo a combination of *Slow* and *Fast* dynamics.

In the following sections, we use the distinct D(q,Δt) characteristics described above to correlate the *Slow*, *Fast*, and *Multimode* classes of dynamics with composite formulation and activity time. Namely, we define the *Slow* class as having D(q,Δt) curves that exhibit single, steady large-Δt plateaus, while *Fast* curves display single large-Δt plateaus but with pronounced oscillations, and the *Multimode* class exhibits two distinct, steady D(q,Δt) plateaus (Fig. 2B).

### Motor competition delays the onset of acceleration and suppresses multimode dynamics

**Fig. 3.**
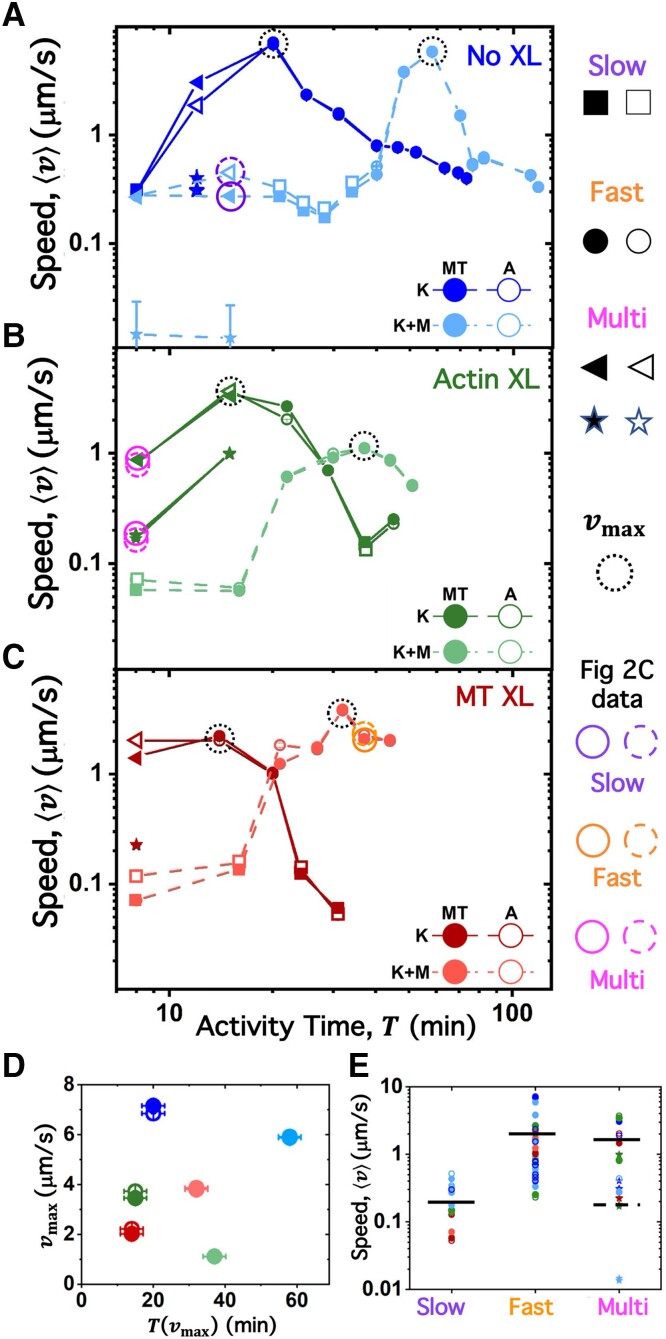
Kinesin-driven composites undergo acceleration and deceleration that are gated by myosin activity and facilitated by crosslinking. (A–C) Speeds ⟨v⟩ of MTs (MT, closed circles) and actin (A, open circles) versus activity time *T* in kinesin-driven composites with no crosslinking (A), actin crosslinking (B), and MT crosslinking (C), without myosin (K, darker shades) and with myosin (K + M, lighter shades). For *Multimode* cases, which have two speeds, the slower speed is indicated by a star. Data points corresponding to the τ(q) curves shown in Fig. 2C are circled in the corresponding color (*Slow* = purple, *Fast* = orange, and *Multimode* = magenta). Error bars (most too small to see) are the larger of the standard error values determined from the Schulz distribution fits and τ(q) distributions (see Methods). (D) Maximum speed $v_{\max}$ reached by each composite, denoted by dashed circles in A, plotted against the time *T* at which $v_{\max}$ occurs. (E) Scatterplot of all 106 actin and MT speeds shown in A–C, divided into *Slow*, *Fast*, and *Multimode* classes. Horizontal lines indicate averages, with the dashed line denoting the average of the slower *Multimode* speeds (stars in A–C).

Having identified quantitative metrics to classify network dynamics, we now determine how the dynamics vary with composite formulation and activity time *T*. We first evaluate the average actin and MT speeds ⟨v⟩ determined from the corresponding τ(q) for each time series (7–15 per formulation) for each of the six composite formulations. Figure 3A–C shows *T*-dependent effects of crosslinking (different panels) and motors (dark versus light shades), with speeds spanning over three orders of magnitude during motor activity. Notably, as suggested by the 2D image structure functions shown in Fig. 2A, actin and MT speeds are well correlated (comparing open and closed symbols) across all composites and activity times despite the varying degrees to which they co-localize or de-mix (Fig. 1B).

We find that actin and MTs in all composites accelerate and reach a maximum speed $v_{\max}$ at activity time $T(v_{\max})$ (Fig. 3D), after which ⟨v⟩ decreases. By classifying each data point in Fig. 3A as *Slow*, *Fast*, or *Multimode* (Fig. 3E), as described above, we measure the average *Slow* speed to be ${\bar{\left\langle v\right\rangle }}_{S}≃0.15$ µm/s, which is an order of magnitude slower than the *Fast* speed of ${\bar{\left\langle v\right\rangle }}_{F}≃1.8$ µm/s. The average low and high speeds for *Multimode* data are comparable to those of *Slow* and *Fast* values, respectively, with ${\bar{\left\langle v\right\rangle }}_{M1}≃0.17$ µm/s and ${\bar{\left\langle v\right\rangle }}_{M2}≃1.7$ µm/s.

We next turn to evaluating how composite formulation programs the different dynamical classes and their dependence on activity time *T*. The average filament speed for the un-crosslinked kinesin-driven composite (no myosin) increases ∼20-fold in the first T≈20 min, transitioning from *Slow* to *Multimode* to *Fast* dynamics (Fig. 3A), reaching $v_{\max}≃7$ µm/s. Following this initial acceleratory period, the composite slowly decelerates over the course of ∼40 min but never returns to dynamics classified as *Slow*. Introducing myosin substantially delays the onset of acceleratory dynamics, increasing $T(v_{\max})$ ∼3-fold, but has little impact on the magnitude of $v_{\max}$ (Fig. 3A and D). Moreover, *Slow* dynamics dominate over more of the activity time than for the kinesin-only composite, as seen by the higher proportion of light blue versus dark blue squares in Fig. 3A.

These results indicate that *Fast* dynamics are due primarily to kinesin-driven motion, as there is minimal change in $v_{\max}$ upon the addition of myosin and that myosin activity counteracts kinesin activity to delay the onset of *Fast* dynamics rather than cooperating synergistically to amplify active dynamics. We can understand this competition as follows.

Keeping in mind that the actin and MTs in the composites form co-entangled interpenetrating networks of comparable mesh sizes, we can assume that every actin filament is sterically interacting with several MTs and other actin filaments and vice versa. Kinesin acts to drive MTs together, which, in turn, attempt to pull co-entangled actin filaments with them, competing with entanglements from other actin filaments that resist kinesin-driven straining. However, because actin filaments are more flexible and relax faster than MTs, they are able to be swept up with the kinesin-driven MT network and then diffuse out of MT-rich clusters to maximize their entropy.

Incorporating myosin into the composites strongly enhances the competition between kinesin-driven pulling of actin and steric entanglements by pulling actin filaments together, which, in turn, attempt to pull interpenetrating MTs with them, counteracting the force of kinesin driving MTs together. The surprising antagonistic interaction between the two motors may also be due to the contractile versus extensile nature of actomyosin and kinesin–MT activity, respectively (62). Namely, kinesin motors are highly processive such that they principally induce nematic bundling, sliding, and extensile motion of rigid MTs, whereas low–duty ratio myosin motors primarily bend, compress, and contract semiflexible actin filaments into asters or foci (49, 57).

We expect this competition to manifest structurally as enhanced actin–MT mixing and interpenetration, as we see in Fig. 1B. In other words, while both filament types are pulled toward like filaments (actin to actin, MTs to MTs) by their respective motors, entanglements with the other filament type resist this motor-driven self-association, thereby facilitating mixing. The net result is reduced clustering and increased actin–MT interpenetration in double-motor composites. While the dynamics eventually mirror those of kinesin-only composites, the structure remains more homogeneous, as shown in Fig. 1B.

The fact that motor antagonism leads to a time delay rather than suppression of active dynamics suggests that eventually kinesin straining beats out myosin straining such that the dynamics mirror kinesin-only composites after being gated by myosin activity. Kinesin–MT straining likely “beats out” actomyosin activity due to the higher density of kinesin clusters compared to myosin minifilaments. As we describe in Methods, in all double-motor composites, there are ∼75 force-generating kinesin clusters for every myosin II minifilament, and the average spacing between kinesin clusters connecting a pair of MTs is ∼12 nm compared to ∼2.6 µm (>200× longer) for myosin minifilaments along actin filaments. This increased density of strain-generating linkers along MTs, as well as their higher duty ratio and processivity, likely causes kinesin–MT force generation to dominate over that of actomyosin.

We now turn to the effect of passive crosslinking on single-motor and double-motor composites. As shown in Fig. 3A, the signatures of motor competition and activity gating seen for unlinked networks are preserved upon crosslinking of both actin (Fig. 3B) and MTs (Fig. 3C). The primary effect of crosslinking is a decrease in the maximum speed $v_{\max}$ and the time over which the composites accelerate to this maximum $T(v_{\max})$ (Fig. 3D). Further, both crosslinking types exhibit *Multimode* dynamics at the onset of activity (red and green triangles and stars), effectively eliminating the initial *Slow* dynamics seen in unlinked composites, likely due to crosslinking reducing the degrees of freedom and increasing the connectivity of the composites, thereby suppressing spatially uncorrelated microscale fluctuations. In other words, large-scale restructuring (attributed to *Multimode* dynamics) and acceleration to $v_{\max}$ are facilitated by crosslinking in kinesin-only composites. Conversely, crosslinking of double-motor composites eliminates the initial *Multimode* dynamics seen for their unlinked counterparts, instead switching directly from *Slow* to *Fast* dynamics with minimal structural reconfiguration. This reduced restructuring of crosslinked double-motor composites compared to kinesin-only composites can be seen in Fig. 1.

To further understand the nature of *Slow*, *Fast*, and *Multimode* dynamics and why crosslinking alters the propensity to exhibit each type, we return to our Fig. 1 results, which show that cross-linking leads to larger and denser filament aggregates compared to unlinked cases. The reduced degrees of freedom and enhanced connectivity that crosslinking provides may explain this enhanced mesoscale clustering, which, in turn, suppresses microscale fluctuations available to the more randomly distributed and less bundled filaments that emerge in the unlinked cases. This mechanistic picture suggests that *Fast* dynamics are dominated by coordinated motion or flow of the composites while uncorrelated microscale fluctuations describe *Slow* dynamics. Conversely, as we describe above, we expect *Multimode* dynamics to arise from mesoscale restructuring, bundling, and de-mixing events.

### Fast, slow, and multimode classes correlate with distinct velocity fields and distributions

To corroborate the mechanisms that we postulate underlie the different dynamical classes in the preceding section, we evaluate the directionality and spatiotemporal coordination of the local dynamics that correspond to the sample *Fast*, *Slow*, and *Multimode* data that we analyze in Fig. 2.

**Fig. 4.**
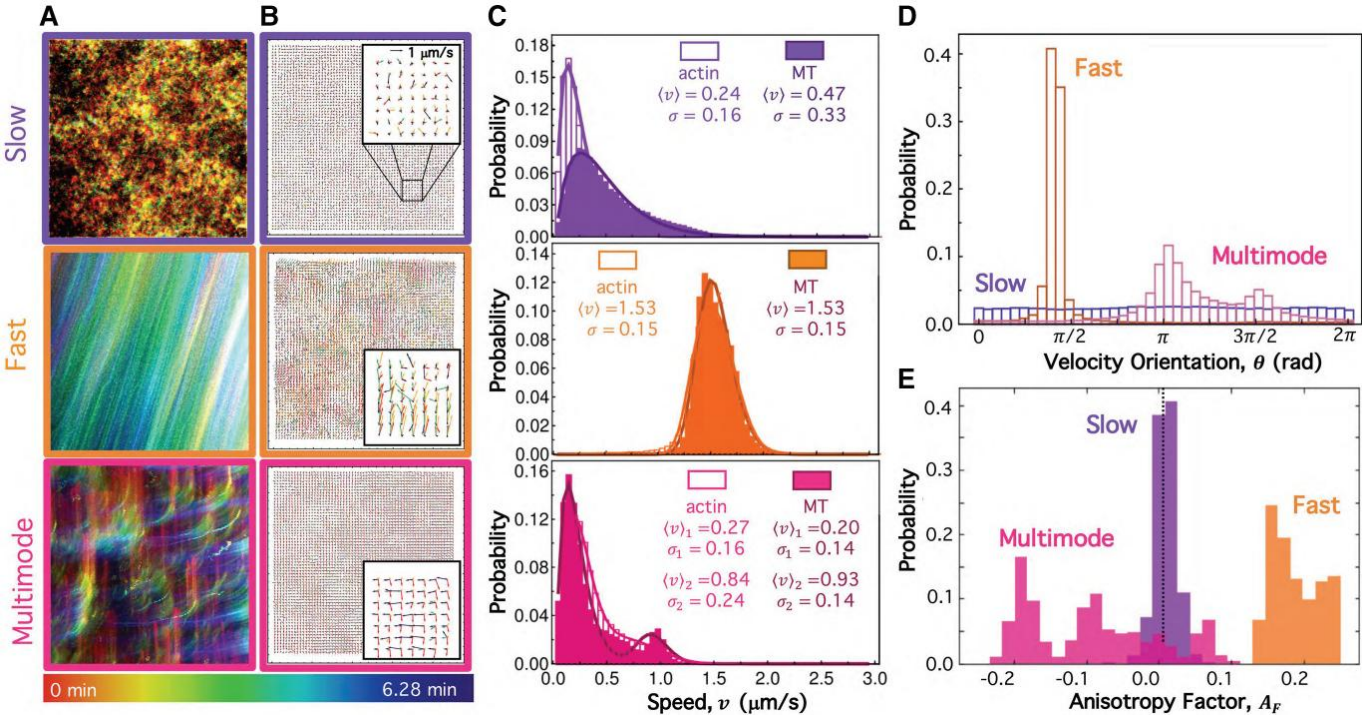
*Slow*, *Fast*, and *Multimode* dynamics classified via DDM exhibit distinct PIV velocity fields and distributions. (A) Temporal color maps, which colorize the features in each 213 µm × 213 µm frame according to the time *t* that the frame is captured during the video, as indicated by the color scale (t=0 min [red] to t=6.28 min [purple]), depict the motion of the composites. *Slow* (top), *Fast* (middle), and *Multimode* (bottom) color maps correspond to the actin channel of the representative videos analyzed in Fig. 2. (B) PIV, performed on the videos analyzed in A, determines corresponding velocity vector fields in which each arrow represents the average velocity $\vec{v}(t)\;$ for an 8 × 8 square-pixel region of the 213 µm × 213 µm field. PIV vector fields for t=0 s (red), 125 s (yellow), 251 s (green), and 377 s (purple) are overlaid in each panel. Insets are zoom-ins of 25 µm × 25 µm regions, as indicated in the top panel. (C) Probability distributions of speeds, determined via PIV, across all vectors in all frames of the actin (open bars) and MT (filled bars) channels of the videos analyzed in B. The solid lines are fits of each distribution to a Schulz distribution function, which describes the functional form of speed distributions assumed in DDM analysis. The average speed ⟨v⟩ and standard deviation σ determined from each fit are listed in the corresponding panel in units of µm/s. The *Multimode* distribution (bottom panel, magenta) is fit to a sum of two Schulz distributions with different ⟨v⟩ and σ values listed in the panel. (D) Probability distributions of the velocity orientations that correspond to the *Slow* (purple), *Fast* (orange), and *Multimode* (magenta) actin speed distributions shown in C. (E) Probability distributions of anisotropy factors $A_{F}$ computed from instantaneous DDM image structure functions$\;D_{i}$ for the same data analyzed in A–D. Dashed vertical line at $A_{F}=0$ indicates isotropic dynamics, whereas $A_{F}>0$ and $A_{F}<0$ correspond to motion in the *y*-direction (∼π/2) and *x*-direction (∼π), respectively.

We first create temporal color maps, which colorize each frame by the time it occurs during the video *t*, and overlay all colorized frames (Fig. 4A; Fig. S3). In this way, the maps depict the motion of the composites over the course of each video. Figure 4A shows the color maps for the actin channel, which are nearly indistinguishable from the MT channel of the same video (see Fig. S3), in line with our DDM results that show that actin and MTs within any given composite exhibit similar dynamics (Figs. 2 and 3). The *Slow* map (top panel) shows small-scale, randomly-oriented motion, while the *Fast* map shows spatially coordinated and nearly unidirectional motion. The *Multimode* map displays features of both *Fast* and *Slow* dynamics, exhibiting directionality on small scales but largely uncorrelated motion at larger scales.

To quantify the features described above, we perform PIV on the actin and MT channels of the videos analyzed in Fig. 4A. PIV vector fields in Fig. 4B and Fig. S4 show overlaid velocity fields at four equally spaced times *t* over the course of the videos analyzed in Fig. 4A. Arrow lengths and directions represent the average velocity of features over 20 frames (∼7.5 s) in the surrounding 8 × 8 square-pixel (6.7 µm × 6.7 µm) region of the field of view.

As shown in Fig. 4B and Fig. S4, *Slow* fields exhibit motion that is slow (small arrows) and randomly oriented (no preferred arrow direction), while *Fast* fields show rapid directional motion with large arrows that all point in a similar direction. *Multimode* fields (Fig. 4B, bottom row) reveal features of both *Slow* and *Fast* modes, as seen by the different arrow sizes and directions. Figure 4C, which shows the histograms of speeds computed from PIV analysis of each video, corroborates the dynamics we observe in the sample flow fields (Fig. 4B) as well as in our DDM analysis (Fig. 2C). Namely, the speed distribution for the *Fast* class (middle row) is shifted substantially to the right of that for the *Slow* video (top row), and the *Multimode* distribution (bottom row) shows two distinct peaks that approximately align with *Slow* and *Fast* distributions, respectively. To further quantify the speed distributions and compare to our DDM results, we fit each histogram to a Schulz distribution (Fig. 2C, solid lines; Fig. S5), which we likewise used in the fitting function for the corresponding DDM image structure functions (see Supplementary Material Methods). We find that the *Slow* and *Fast* distributions are well described by a single Schulz distribution, while *Multimode* distributions require a sum of two Schulz distributions. The average speed ⟨v⟩ and standard deviation σ determined from each fit (listed in the corresponding panel) show that the speeds measured in Fourier space using DDM (Fig. 2B) and in real space using PIV are statistically indistinguishable (Table S2), with average values of ${\bar{\left\langle v\right\rangle }}_{S}\approx 0.3\;\mu m/s$, ${\bar{\left\langle v\right\rangle }}_{F}\approx 1.7\;\mu m/s$, ${\bar{\left\langle v\right\rangle }}_{M1}\approx 0.2\;\mu m/s$, and ${\bar{\left\langle v\right\rangle }}_{M2}\approx 0.8\;\mu m/s$ for *Slow* (S), *Fast* (F), and *Multimode* (M1, M2) videos, respectively.

Motivated by the apparent class-dependent anisotropy (or lack thereof) in the PIV vector fields, we also evaluate the corresponding velocity orientation distributions (Fig. 4D), which reveal isotropic motion for the *Slow* class, with no perceptible peak and comparable occurrences of all angles, compared to sustained unidirectional *Fast* motion, as evidenced by the sharply peaked narrow distribution. The *Multimode* distribution displays features of both *Fast* and *Slow* distributions, with a broader sampling of directions compared to *Fast* but with more pronounced peaks compared to *Slow*.

As noted in the previous section, we also see evidence of anisotropic dynamics in our DDM analysis, manifested as radial asymmetry in the $D(q_{x},q_{y},\Delta t)$ plots for the *Fast* class and to a lesser extent in the *Multimode* plots (Fig. 2A; Fig. S2). To quantify this anisotropy in Fourier space, we evaluate an anisotropy factor $A_{F}(q,t)$ by computing weighted azimuthal integrals of the DDM image structure function (detailed in Methods and Supplementary material (63)). $A_{F}$ can assume values between −1 and 1 for *x*- and *y*-directed motion, respectively, with $A_{F}=0$ indicating isotropic motion. Figure 4E shows that the distributions of $A_{F}$ values for *Slow* and *Fast* classes exhibit distinct peaks at $A_{F}\approx 0$ and $A_{F}>0$, indicative of isotropic and *y*-oriented motion, respectively. Conversely, the *Multimode* distribution is broader with multiple peaks that span from $A_{F}<0$ to $A_{F}>0$ and include a significant fraction of near-zero values. Likewise, the *Multimode* PIV orientation distribution samples a broad range of angles (isotropic, $A_{F}=0$) while also exhibiting distinct peaks (directionality, $|A_{F}|>0$).

### Spatiotemporal variations in dynamics are suppressed by motor antagonism

To better elucidate the mechanisms dictating the different dynamical classes and the influence of motor antagonism on said mechanisms, we use both DDM and PIV to resolve variations in the short-time dynamics of the composites, i.e. those that occur within the time *t* of a given video.

**Fig. 5.**
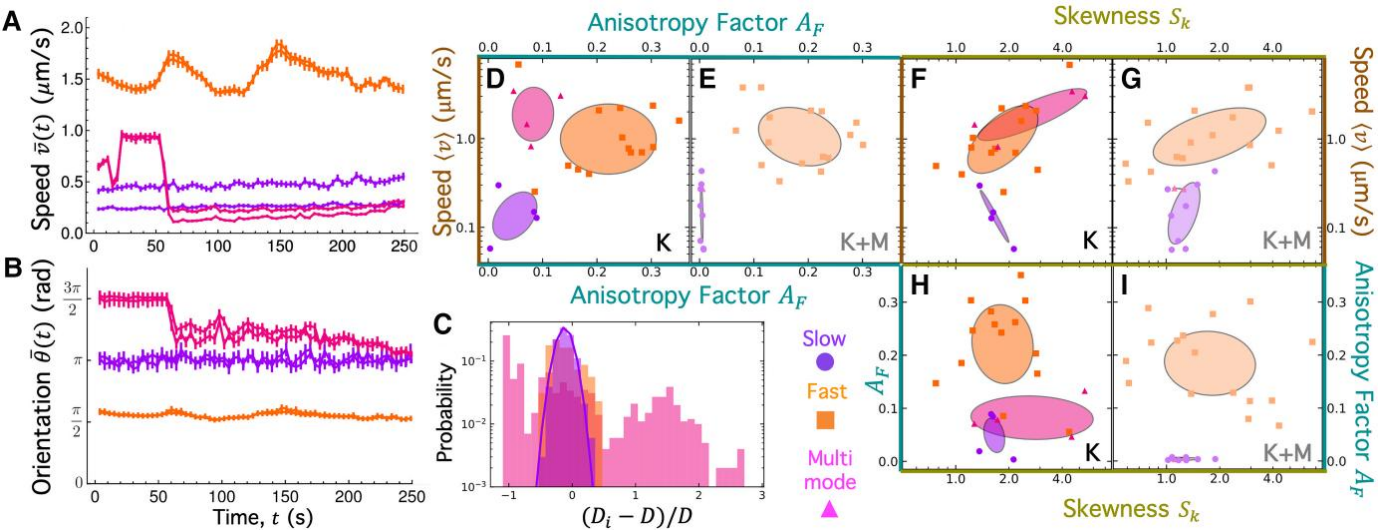
Non-stationary fast dynamics, unique to the *Multimode* class, indicate discrete intermittent restructuring. (A) Average speed $\bar{v}(t)$ versus time *t* measured via PIV for the actin and MT channels of the representative *Slow* (purple), *Fast* (orange), and *Multimode* (magenta) videos analyzed in Fig. 4. $\bar{v}(t)$ for each time *t* is an average over all vector magnitudes in the PIV flow field associated with time *t*. (B) Average velocity orientations $\bar{\theta }(t)$ versus *t* computed from the same vector fields following the same method as in A. (C) Probability distributions of instantaneous image structure function values, $D_{i}(q,\Delta t,t)$, over all *t* for $q=0.30\;\mu m^{-1}$ and Δt≤3.8s computed for the videos evaluated in A and B. To better compare the probability distributions for the different classes, $D_{i}$ is normalized by the *t*-averaged image structure function $D=\;\langle D_{i}\rangle_{t}$. The solid purple line is a fit of the *Slow* distribution to a Gaussian function. Deviation from Gaussianity indicates sporadic discrete structural changes and is quantified by skewness $S_{K}$. (D–I) Stacked three-dimensional confidence ellipse plots show the relationships between average speed *v* (D–G; brown axes), anisotropy factor magnitude $\left|A_{F}\right|$ (D, E, H, and I; teal axes), and skewness $S_{K}$ (F–I; gold axes). Data points, with colors and symbols indicating dynamical class according to the legend, correspond to the 106 data points plotted in Fig. 3, and the ellipses enclose one standard deviation around the mean. Panels with darker shaded (D, F, and H) and lighter shaded (E, G, and I) ellipses display data for composites with kinesin (K) and both kinesin and myosin (K + M), respectively.

We first evaluate the average speed $\bar{v}(t)\;$as a function of time *t* for the actin and MT channels of each video analyzed in Figs. 2 and 4, which we compute from the corresponding PIV vector fields, where $\bar{v}$ is averaged over all vectors in a single field. As shown in the $\bar{v}(t)\;$plots in Fig. 5A and Fig. S6, *Slow* and *Fast* dynamics are largely stationary over the course of a given video, with nearly constant speeds. In contrast, *Multimode* traces show discrete and abrupt shifts from intermediate to fast motion to steady slow motion.

Observing the time dependence of the corresponding average velocity orientations $\bar{\theta }(t)$, we find similar trends as for $\bar{v}(t)$, whereby the directionality of both *Fast* and *Slow* examples is nearly independent of *t*, while the average orientation of *Multimode* vectors undergoes an abrupt and discrete shift at t≃60 s.

To corroborate and better characterize the apparent stationarity of *Slow* and *Fast* class dynamics and the non-stationary *Multimode* dynamics shown in Fig. S5A and B, we compute instantaneous DDM image structure functions $D_{i}(q,\Delta t,t)$, which, unlike the D(q,Δt) curves shown in Fig. 2B, are not averaged over time *t* (63). By evaluating the probability distribution of $D_{i}(q,\Delta t)$ values for all *t* in a given video, we can determine the extent to which dynamics are temporally heterogeneous during the acquisition time. Namely, ergodic stationary dynamics are expected to follow a Gaussian distribution of structural correlations, which are quantified by $D_{i}(q,\Delta t,t$). As shown in Fig. 5C, the *Fast* and *Slow* distributions are strongly overlapping, with the *Slow* distribution being well fit to a Gaussian function. Conversely, the *Multimode* distribution is distinctly non-Gaussian—with no obvious peak, a broad distribution of values, and significant noise—indicative of large intermittent fluctuations in structural correlations (63).

To quantify the extent to which the temporal $D_{i}\;$distributions deviate from Gaussianity, we compute the skewness $S_{K}=(\langle D_{i}-D\rangle {)}^{3}/(\langle (D_{i}-D{)}^{2}\rangle {)}^{3/2}$, which is zero for a Gaussian distribution. For reference, the distributions shown in Fig. 5C have skewness values of $S_{K,S}≃0.42$, $S_{K,F}≃0.66$, and $S_{K,M}≃0.86$ for the *Slow*, *Fast*, and *Multimode* classes, respectively. Positive skewness, largest for *Multimode* distributions, has been reported for colloidal gels that are en route toward arrest and has been interpreted as arising from discrete restructuring processes such as coalescing or rupturing, as well as intermittent fluctuations and rearrangements (63).

To determine the prevalence of non-stationary dynamics across the formulation phase space and activity times, we compute skewness values for all composite formulations and times *T* evaluated in Fig. 3. Figure 5D–I shows stacked confidence ellipse plots comparing skewness $S_{K}$, average speeds ⟨v⟩, and anisotropy factors $A_{F}$ colorized by dynamical class and separated into panels for kinesin-driven composites without (Fig. 5D, F, and H) and with (Fig. 5E, G, and I) myosin (also see Fig. S7). The individual points correspond to all data points shown in Fig. 3, and the ellipses enclose one standard deviation around the mean. As shown, the *Multimode* data exhibit the largest skewness values, as seen by the magenta ellipses being furthest to the right in Fig. 5F and H. *Fast* and *Slow*$S_{K}$ values are similar to one another and deviate less from zero. The higher skewness for *Multimode* data is coupled with relatively fast speeds (Fig. 5F) but low anisotropy (Fig. 5H). These couplings further support our interpretation that *Multimode* dynamics arise from large intermittent restructuring events, which we expect to have no preferred directionality but give rise to periods of time—e.g. during a restructuring event—that exhibit fast dynamics.

Comparison of the composites driven by kinesin only (Fig. 5D, F, and H; darker shades) versus two motors (Fig. 5E, G, and I; lighter shades) reveals that the presence of myosin nearly eliminates *Multimode* dynamics, as evidenced by the lack of magenta ellipses in Fig. 5E, G, and I. Further, the distributions of data points for the double-motor composites generally exhibit smaller skewness values as compared to kinesin-only composites, as seen by the ellipses shifted to the left in Fig. 5G and I compared to Fig. 5F and H. Despite these differences, we also observe that the distributions of speeds for composites with and without myosin are not significantly distinct, as we discussed in the previous section (also see Fig. 3).

Taken together, these results demonstrate that *Multimode* dynamics arise from discrete and abrupt restructuring events and coarsening, and the presence of myosin suppresses this restructuring such that double-motor composites exhibit very few instances of *Multimode* dynamics and remain more homogenously mixed at the end of the activity. In the absence of mesoscale discrete restructuring, the double-motor networks take longer to coarsen and switch to *Fast* coordinated flow.

### Motor competition inhibits composite restructuring and de-mixing enhanced by crosslinking

To connect the dynamics we measure with various structures and reconfiguration, we develop a minimal model that aims to capture the key dynamical features of our composites. As described in Methods and Supplementary material, our model simulates filament motion that arises from motor-driven advection and thermal diffusion and works against steric hindrances and viscous traps due to motor and protein crosslinking (see Fig. S8 and Table S1). We purposefully simplify the model, ignoring details such as filament flexibility and individual motor dynamics that other models incorporate (64–66), to facilitate applications to other systems and identify the key parameters that dictate the experimental phenomena we observe.

**Fig. 6.**
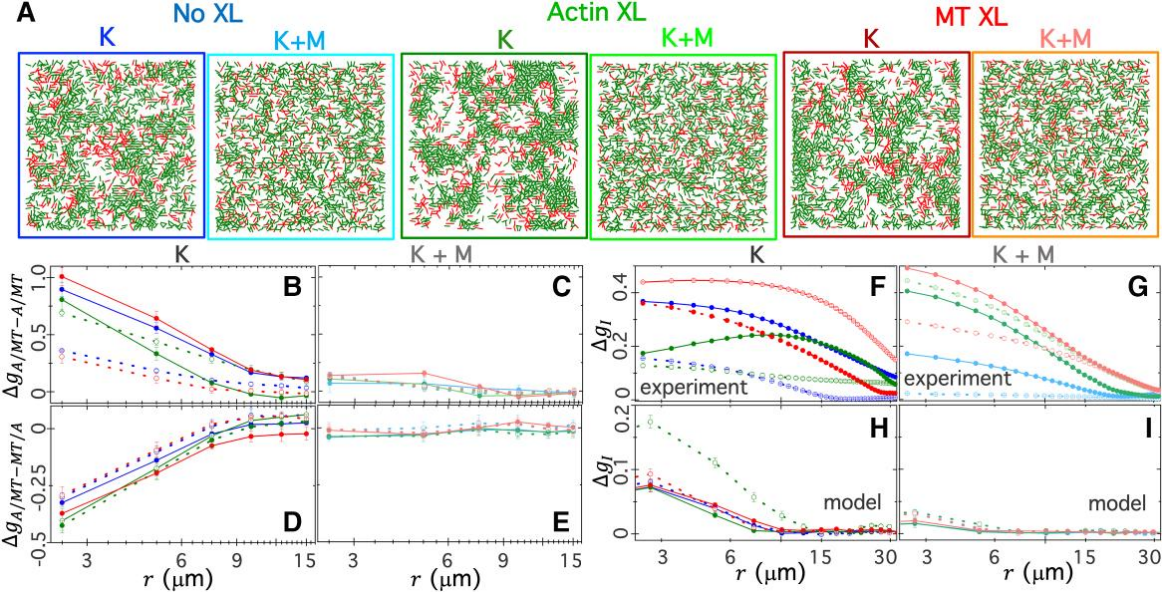
Minimal advection–diffusion modeling characterizes and corroborates expected motor-driven restructuring. (A) Simulation snapshots, each 155μm×155μm, show sample final configurations of MTs (red) and actin (green) in composites with no passive crosslinking (No XL), actin crosslinking (Actin XL), and MT crosslinking (MT XL), subject to active forcing by kinesin (K) or both kinesin and myosin (K + M). Simulation details are provided in Supplementary Material Methods and Table S1. (B–I) The difference between the initial (T=0) and final ($T=T_{F}$) values of various structural correlation functions, $\Delta g_{\_}(r)=g_{\_}(r,\;T_{F})-g_{\_}(r,\;0)$, versus radial distancer between two filaments (B–E) or pixels (F–I) for composites depicted in A. The type of passive crosslinking is color coded according to the border colors in A. (B, C) The difference in like-filament distributions for actin ($\Delta g_{A-A}$, open symbols) and MTs ($g_{\mathrm{MT}-\mathrm{MT}}$, filled symbols) in composites with (B) kinesin or (C) both kinesin and myosin. (D, E) Unlike-filament distribution differences for (D) actin ($g_{A-\mathrm{MT}}$, open symbols) and (E) MTs ($g_{\mathrm{MT}-A}$, filled symbols) for the composites analyzed in B and C. (F–I) SIA differences $\Delta g_{I}$ for actin (open symbols) and MTs (filled symbols) computed from (F, G) experimental time-series and (H, I) simulation snapshots for composites with (F, H) kinesin or (G, I) both kinesin and myosin. Error bars for simulations are standard errors across three replicates and for experiments are standard errors across 100 images from three time-series.

Our model simulations show that all composites start as homogeneous interpenetrating networks of actin and MTs at T=0 (Fig. S9), as we see in experiments (Fig. 1B), but subsequently restructure to varying degrees depending on the composite formulation. Figure 6A, which shows sample simulation snapshots of the final states ($T=T_{F}$) of the six composite formulations, reveals strong suppression of restructuring by motor competition, similar to our experimental observations, with the K + M composites undergoing substantially less restructuring and de-mixing than the kinesin-only composites. Also, in line with experiments, crosslinking of either actin or MTs in simulated composites enhances aggregation and clustering compared to composites without crosslinkers. This agreement between model predictions and experimental observations suggests that it is the balance between frictional jamming and motor-driven de-mixing that dictates the different formulation-dependent structural regimes.

To quantify the degree of restructuring in simulations, we compute the probability distributions of like filaments ($g_{A/\mathrm{MT}-A/\mathrm{MT}}(r)$) and unlike filaments ($g_{A/\mathrm{MT}-\mathrm{MT}/A}(r)$), a radial distance *r* from a given actin/microtubule (A/MT) for the initial (T=0) and final $(T=T_{F}$) states of all composites (see Supplementary Material Methods). For homogeneous well-mixed networks, all distributions should equate to 1 for all *r* values, which we find to be the case for the initial states of all simulated composites (Fig. S9). The more $g_{A-A}(r)$ or $g_{\mathrm{MT}-\mathrm{MT}}(r)$ values are above 1, the more clustering of actin or MTs, respectively. Conversely, $g_{A-\mathrm{MT}}(r)<1$ or $g_{\mathrm{MT}-A}(r)<1$ indicates segregation and de-mixing of actin from MTs or vice versa. Figure 6B–E plots the differences between the final and initial distributions, e.g. $\Delta g_{A-A}=g_{A-A}(r,T_{F})-g_{A-A}(r,0)$, such that values of zero indicate minimal restructuring, while positive “like” distributions and negative “unlike” distributions indicate like-filament clustering and de-mixing of unlike filaments, respectively. As shown, the composites with kinesin and myosin show minimal de-mixing regardless of crosslinking (Δg≈0 in Fig. 6C and E), while all composites without myosin show signatures of clustering and de-mixing, which is generally more pronounced in the crosslinked composites (Fig. 6B and D).

Finally, to directly compare the predicted and experimental restructuring, we perform identical spatial image autocorrelation (SIA) analysis (see Methods) on the initial and final experimental videos and simulation snapshots. SIA computes the correlation in intensities $g_{I}(r)$ between two pixels separated by a radial distance *r* in a given image such that $g_{I}(r)$ indicates the lengthscales over which structural features in an image are correlated. Specifically, $g_{I}(r)$ values range from 1 for complete correlation (such as when r=0) to 0 for complete decorrelation, e.g. for *r* values much larger than the size of structural features. Similar to Fig. 6B–E, we evaluate the differences between the final and initial correlation functions $\Delta g_{I}(r)$ for actin and MTs in all simulated composites (Fig. 6H and I), which we compare to experimental values (Fig. 6F and G). We find that in both experiments and simulations, the presence of myosin reduces the distance over which structural correlations are enhanced over the time course of motor activity, evidenced as faster decay in $\Delta g_{I}(r)$ with increasing *r* in Fig. 6G and I compared to Fig. 6F and H. This feature is indicative of reduced large-scale clustering and de-mixing of actin and MTs, as is also evident in Figs. 1B and 6A. Moreover, in both experiments and simulations, passive crosslinking generally leads to increased structural correlations (larger $\Delta g_{I}$ values) compared to composites without crosslinkers, in particular at larger distances and for actin crosslinking. The increased aggregation with actin crosslinking manifests in experiments as minimal decay and non-monotonic dependence of $\Delta g_{I}(r)$ with increasing *r* for actin and MTs, respectively, indicative of fewer small-scale clusters and increased mesoscale (>10 µm) structural correlations. In simulations, increased aggregation can be seen as larger $\Delta g_{I}$ values in the presence of actin crosslinkers across all lengthscales.

We note that given the simplicity of our model and the simulated renderings of the composites, as well as the noise in our microscope images, as can be seen in Fig. 1 and Movies S1–S3, we do not expect quantitative agreement between experiments and simulations. Rather, we aim to capture qualitatively similar dependence of structural features on crosslinking and motor competition, as we describe above. Namely, the presence of myosin inhibits restructuring while passive crosslinking enhances it. The generally larger $\Delta g_{I}$ values measured in experiments compared to simulations are likely due to the noise and finite depth of the experimental images, which limit the occurrence of “empty space” that are seen in simulated composites, thereby overestimating correlations across lengthscales as compared to simulated images. Moreover, the flexibility of the actin filaments, not accounted for in the model, may also allow for greater restructuring and clustering.

To understand the underlying mechanisms driving this restructuring more fully, we consider that while kinesin motor activity adds to the advective term for MTs in the model, the processive nature of kinesin also increases the drag on the MTs. Conversely, the addition of non-processive myosin motors increases filament advection with a relatively smaller increase in drag. Thus, kinesin activity acts to collect MTs into locally arrested clusters that can either sweep up or squeeze out actin filaments. The addition of passive crosslinking of actin or MTs accelerates this process by facilitating the coalescence of smaller clusters into larger ones. On the other hand, myosin activity allows for filament redistribution within clusters and diffusive migration of filaments out of clustered regions, thereby inhibiting segregation between actin and MTs and increasing the rate at which newly formed clusters can dissolve back into a mixed state. Succinctly stated, motor antagonism can arise from an interplay between competitive motor-driven advection and frictional drag, irrespective of its origin—steric interactions or passive or active crosslinking.

## Conclusion

The cytoskeleton is a non-equilibrium multifunctional composite comprising diverse protein filaments, motors, and crosslinkers that cooperate and compete to enable diverse cellular structures and processes. As such, the cytoskeleton is one of the primary inspirations to the burgeoning field of active matter, and much of current active matter research seeks to learn from and emulate the cytoskeleton. The composite nature of the cytoskeleton, which confers its signature versatility and programmability, is one of its hallmarks. Yet, current active matter platforms are largely limited to a single force-generating component and/or substrate. We address this gap by engineering co-entangled and crosslinked composites of MTs and actin filaments driven by kinesin and myosin motors—breaking new ground in active matter design by incorporating multiple independently tunable force-generating components and active substrates.

By coupling Fourier-space and real-space analyses (DDM and PIV), we show that composites undergo a combination of *Fast* advective flow, *Slow* isotropic fluctuations, and *Multimode* restructuring that result in structures ranging from interpenetrating actin–MT scaffolds to de-mixed amorphous clusters. Surprisingly, competition between kinesin and myosin straining delays the onset of kinesin-driven acceleration without appreciably changing the range of speeds that the different composites exhibit. Motor antagonism also suppresses mesoscale restructuring events that underlie *Multimode* dynamics, thereby sustaining mixed networks of actin and MTs. Conversely, passive crosslinking hastens the onset of kinesin-mediated acceleration and subsequent deceleration by enhancing network connectivity and suppressing uncorrelated microscale motion. Importantly, the emergent dynamics and extensive programmable phase space of non-equilibrium properties we reveal are a result of very subtle changes in substrate connectivity and activity.

Our work brings reconstituted cytoskeleton systems an important step closer to mimicking the complexity of the active composite cytoskeleton by integrating two distinct and ubiquitous motor–filament systems, actomyosin and kinesin–MT networks, that have been shown to interact and co-mediate important cellular processes including morphogenesis and exocytosis (67, 68), mechanosensation (69), and migration and stiffening (70). Interactions between actomyosin, kinesin, and MTs have also been implicated in wound healing, mitosis, and cytoplasmic streaming (7, 15, 16, 28, 43, 71, 72). Because the motor and filament concentrations in our composites are within physiological ranges (73), our results offer new insight into the macromolecular dynamics and interactions that contribute to these cellular processes. We note that cell-like confinement of in vitro cytoskeletal networks has also been shown to play a key role in recapitulating dynamics and structures seen in cells (71, 72, 74). We plan to build in this layer of complexity in our future work (75).

Finally, the programmability of our composites, with multiple well-controlled tuning knobs—motors, filaments, and crosslinkers—which can each be varied independently while maintaining composite integrity, opens the door for reconfigurable materials that can be programmed to exhibit varying types and rates of motion and restructuring over broad spatiotemporal scales. For example, materials based on our designs could be used as spatially controlled microactuators, responsive filtration and sequestration devices, and self-curing and self-repairing infrastructure technologies. Our minimal advection–diffusion model that recapitulates our experimental trends is broadly applicable to active composite networks and lays the foundation for more complex predictive models that quantitatively capture the structure and dynamics of composite active matter. As such, we anticipate that our double-motor material design, intriguing dynamical results, and corresponding modeling framework will spark a new class of studies that explore the broad parameter space of this platform.

## Methods

See Supplementary Material Methods for more detailed descriptions of each of the following sections.

### Protein preparation

Rabbit skeletal actin monomers (Cytoskeleton), biotinylated actin monomers (Cytoskeleton), porcine brain tubulin dimers (Cytoskeleton), biotinylated tubulin dimers (Cytoskeleton), rhodamine-labeled tubulin dimers (Cytoskeleton), and myosin II (Cytoskeleton) are reconstituted and flash-frozen into single-use aliquots according to previously described protocols (28, 48). Biotinylated kinesin-401 is expressed in Rosetta (DE3)pLysS competent *E. coli* (Thermo Fisher) and purified, as described in Supplementary material.

For composites that incorporate actin or MT crosslinking, actin–actin or MT–MT crosslinker complexes are prepared according to previously described protocols (48). In brief, biotinylated actin or biotinylated tubulin is combined with NeutrAvidin and free biotin at a ratio of 2:2:1 protein:free biotin:NeutrAvidin.

Immediately prior to experiments, myosin II is purified as previously described (27) and stored at 4°C. Kinesin clusters are formed by incubating dimers at a 2:1 ratio with NeutrAvidin with 4 µM DTT for 30 min at 4°C.

### Active cytoskeleton composite preparation

Actin-MT composites are formed by polymerizing 2.32 μM unlabeled actin monomers and 3.48 μM tubulin dimers (5% rhodamine-labeled) in PEM-100 (100 mM PIPES, 2 mM MgCl_2_, and 2 mM EGTA) supplemented with 0.1% Tween, 10 mM ATP, 4 mM GTP, 5 μM Taxol, and 0.47 μM AlexaFluor488–phalloidin (Life Technologies).

For crosslinked composites, a portion of either actin monomers or tubulin dimers is replaced with equivalent crosslinker complexes to achieve the same overall actin and tubulin concentrations and crosslinker:protein ratios of $R_{A}$ = 0.02 for actin or $R_{\mathrm{MT}}$ = 0.005 for MTs. $R_{A}$ and $R_{\mathrm{MT}}$ values are chosen to achieve similar lengths between crosslinkers *d* along actin filaments and MTs ($d_{A}≃\;60$ nm, $d_{\mathrm{MT}}≃\;67$ nm) (48) and to be high enough to induce measurable changes in the viscoelasticity compared to unlinked networks but low enough to prevent filament bundling.

Composites are polymerized for 30 min at 37°C, after which 1.86 μM phalloidin is added and the composite is incubated for 10 min at room temperature. Following, 50 μM blebbistatin (26), an oxygen scavenging system, 0.47 μM myosin II, and 0.35 μM kinesin are added. Concentrations of actin, tubulin, myosin II, and kinesin are within reported physiological ranges (73, 76, 77).

While myosin activity is controlled by blebbistatin deactivation, kinesin starts to act immediately, so T=0 for each experiment is set as the time kinesin is added. Each sample is gently flowed into a ∼1 mm × 24 mm sample chamber composed of a silanized (78) coverslip and microscope slide fused together by a ∼100-μm-thick parafilm spacer and sealed with epoxy, creating an airtight chamber.

### Fluorescence microscopy

Imaging of AlexaFluor488-labeled actin and rhodamine-labeled MTs comprising composites is performed using a Nikon A1R laser scanning confocal microscope with a 60× 1.4 NA oil immersion objective (Nikon), 488-nm laser with 488/525-nm excitation/emission filters, and 561-nm laser with 565/591-nm excitation/emission filters. 488-nm illumination also locally deactivates blebbistatin (26–28). Time-series (videos) of 256 × 256 square-pixel (213 μm × 213 μm) images are collected at 2.65 fps for a maximum video time of $t_{\max}=1000$ frames (∼377s≃6.28min). Imaging begins 5 min after the addition of kinesin motors (*T* = 5 min) in the middle of the ∼100-μm-thick sample chamber. Each successive video is collected in a different field of view of the same sample until there is no longer any discernible restructuring or motion (T≃60−120min). A total of 7–15 videos are collected for each of the six formulations (no crosslinking, actin crosslinking, and MT crosslinking, with and without myosin). Each video includes two channels that separate the actin and MT signals such that they can be processed separately and compared.

### DDM

DDM is performed on the actin and MT channels of each video, as described previously (28). Image structure functions are determined by taking the square of 2D Fourier transforms of differences between an image at time *t* and one at t+Δt. This process yields the instantaneous image structure function $D_{i}(q_{x},q_{y},\Delta t,t)$, where $q_{x}$ and $q_{y}$ are *x* and *y* components of the wave vector $\vec{q}$. As typically done in DDM analysis, we average $D_{i}$ over all times *t* (frames) of a given video, and all wave vectors $\vec{q}$ with the same magnitude *q*, to determine the 1D image structure function D(q,Δt) that can be fit to various models. We fit D(q,Δt) versus Δt for each wave vector *q* to a model in which the distributions of speeds are described by one or two Schulz functions (60) (see Supplementary Material Methods), as has been done to describe other ballistic biological systems (60, 61). For *Slow* and *Fast* data in which one distribution is sufficient, there are four free parameters (A, *B*, $\tau_{1}$, $Z_{1})$, whereas for *Multimode* data, there are seven (adding $\tau_{2}$, $Z_{2}$, and *f*) (see Supplementary Material Methods). For each video, we perform fits for 40 different *q* values in the range *q* = 0.8−2 $\mu m^{-1}$ (∼3–8 μm), from which we extract τ(q) curves for the actin and MT channels. By fitting each τ(q) curve to $\tau (q)=(\langle v\rangle q{)}^{-1}$, we compute the average speed ⟨v⟩ for each channel of each video. We determine the error associated with ⟨v⟩ using two methods. First, we compute *v* from each individual (τ, *q*) pair (i.e. v=1/τq) and determine the standard error across those values. Second, we use the Schulz parameter *Z* determined from our D(q,Δt) fits and our measured ⟨v⟩ value to compute the standard deviation σ and corresponding standard error via the relation $Z={\left(\frac{\left\langle v\right\rangle }{\sigma }\right)}^{2}-1$. Error bars shown in Fig. 3 are the larger of the two values for each case.

To determine the degree to which dynamics deviate from radial symmetry, implying directionality, we compute the anisotropy factor $A_{F}\;$ of $D_{i}(q_{x},q_{y},\Delta t,t)$ in *q*-space by computing $A_{F}(q,\Delta t,t)=\overset{2\pi }{\underset{0}{\int }}\;D(q,\Delta t,\theta )\cos\;(2\theta )d\theta /\overset{2\pi }{\underset{0}{\int }}\;D(q,\Delta t,\theta )d\theta$ and averaging over *q*, Δt, and *t* (79, 80). θ is defined relative to the positive *y*-axis such that $A_{F}>0\;$ and $A_{F}<0\;$ correspond to motion along the *y*- and *x*-directions, respectively, and $A_{F}=0$ indicates isotropic motion.

To evaluate the time dependence of dynamics over short timescales (within the time *t* of a single video), we investigate the temporal distribution of instantaneous image structure functions $D_{i}(q_{x},q_{y},\Delta t,t)$ for a given Δt and *q*. For steady-state dynamics, one would expect this distribution to be Gaussian. Deviations from Gaussianity indicate sporadic events, which cause larger than typical structural decorrelations. We quantify this non-Gaussian behavior by evaluating the skewness, $S_{K}=(\langle D_{i}-D\rangle {)}^{3}/(\langle (D_{i}-D{)}^{2}\rangle {)}^{3/2}$, where the average is over Δt and *q*.

### PIV

PIV is performed using the GPU-accelerated version of OpenPIV (81). Interrogation windows of 8 × 8 square pixels, with a 4 × 4 square-pixel overlap, are used to generate 64 × 64 grids of velocities for MT and actin channels of each time series. Average velocities $\vec{v}$ for each interrogation window are determined from image pairs separated by Δt= 10 frames (∼3.77 s). From the measured velocities, we determine the distribution of individual speeds v(t) and velocity orientations θ(t) across each image over the course of a video. To identify and exclude spurious velocities during statistical analysis, we reject those points for which the signal-to-noise ratio was <2. We fit the speed distributions to Schulz distributions by minimizing the mean square difference between the predicted statistical weight assigned to each bin (of width 50 nm/s) for a given choice of parameters and the actual fraction of speeds in each bin. Arrows plotted in Fig. 4B and Fig. S4 represent the local velocity on a regular Cartesian grid, with arrow length proportional to speed. Visualizations at different video times *t* are superposed, with arrow color representing *t*.

### SIA

SIA is performed separately on actin and MT channels of microscope images and simulation snapshots (see below) using custom Python scripts (27, 28, 81). SIA measures the correlation in intensity $g_{I}$ of two pixels in an image as a function of separation distance r (82). We generate autocorrelation curves $g_{I}(r)$ by taking the fast Fourier transform of the image F(I), multiplying by its complex conjugate, applying an inverse Fourier transform $F^{-1}$, and normalizing by the squared intensity: $g_{I}(r)=\frac{F^{-1}({\left|F(I(r))\right|}^{2})}{{\left[I(r)\right]}^{2}}$. Correlation curves shown in (i) Fig. 6F and G and (ii) Fig. 6H and I are averages across (i) 100 microscope images from three time series and (ii) simulation snapshots from three independent runs (see below). Error bars indicate the standard error.

### Computational model

To predict motor-driven restructuring, we develop a minimal model that captures the key energetic components of our system, as fully described in Supplementary Material Methods and Table S1. In brief, we allow filaments to interact with neighboring filaments via (i) motor-generated forces that can either pull the interacting filaments toward each other or push them away and (ii) crosslinks that increase the frictional forces on the interacting filaments. The movement of a filament center to a neighboring grid point within a small temporal time step is then a stochastic event with a probability that can be calculated by the standard solution to the Fokker–Planck equation given by $p_{i}(x\geq l)=1-\frac{1}{2}\left(1+\mathrm{erf}\left(\frac{l-\mu_{i}}{\sigma_{i}\sqrt{2}}\right)\right)$, where *l* is the distance to the next grid point in a particular direction, $\mu_{i}$ is the average advection-induced displacement in that direction, and $\sigma_{i}$ is the diffusion-based rms 1D displacement of the filament along the direction to the specific grid point. The subscript *i* represents a specific filament in the model. The movement probability of filament *i* to a neighboring grid point that either (i) contains the center of filament *j* or (ii) is empty is given by (i) $p_{ij}=\;p_{i}\times p_{j}$ or (ii) $p_{ij}=\;p_{i}\times 1$.

We implement our model on a 150 µm × 150 µm hexagonal lattice with 2.5-µm spacing and use numerical values for all model parameters that are based on experimental and literature values (see Table S1). Initially, each lattice point is either empty or occupied by a center of an actin filament or MT using probabilities matching the average volume fraction occupied by these elements. The movement of the filaments is simulated in each iteration by calculating the likelihood of each possible movement $p_{ij}$ for all grid pointsi containing filament centers and randomly picking one of these movements to occur based on these probabilities (Fig. S8) (83). We perform three independent simulation runs for each composite formulation (Fig. S9).

To quantify the degree of clustering and segregation of the different filaments, we compute the probability distributions of filaments that are alike, $g_{A-A}(r)=\;\left\langle \frac{N_{A}(r)}{\;f_{A}N(r)}\right\rangle \;\mathrm{or}\;g_{\mathrm{MT}-\mathrm{MT}}(r)=\;\left\langle \frac{N_{\mathrm{MT}}(r)}{\;f_{\mathrm{MT}}N(r)}\right\rangle$, and unlike, $g_{A-\mathrm{MT}}(r)=\;\left\langle \frac{N_{\mathrm{MT}}(r)}{\;f_{\mathrm{MT}}N(r)}\right\rangle \;\mathrm{or}\;g_{\mathrm{MT}-A}(r)=\;\left\langle \frac{N_{A}(r)}{\;f_{A}N(r)}\right\rangle$, a radial distance *r* from a given actin filament (A) or MT (see Supplementary Material Methods). In the above, $N_{A/\mathrm{MT}}(r)$ is the number of actin/MT neighbors at distance *r* from a specific filament, $f_{A/\mathrm{MT}}$ is the actin/MT volume fraction, and N(r) is the maximum number of possible neighbors that reside a distance rfrom the specific actin/MT. An increase in $g_{A/\mathrm{MT}-A/\mathrm{MT}}(r)$ above 1 indicates clustering of actin/MTs, while a decrease in $g_{A/\mathrm{MT}-\mathrm{MT}/A}(r)$ below 1 indicates segregation of unlike filaments. Correlation analysis data shown in Fig. 6B–E are averages over all filaments of the same type over three statistically independent replicates with error bars representing the standard error.

## Supplementary Material

pgad245_Supplementary_Data

## Data Availability

All data generated or analyzed during this study are included in this published article and its supplementary information files or available at Zenodo: doi:10.5281/zenodo.8165779.
