# Supplementary material for: Kinesin and myosin motors compete to drive rich multiphase dynamics in programmable cytoskeletal composites
Source: PNAS Nexus. 2023 Jul 31;2(8):pgad245. doi: 10.1093/pnasnexus/pgad245 (PMC10416814; doi:10.1093/pnasnexus/pgad245)
Supplement: pgad245_Supplementary_Data [file pgad245_supplementary_data.zip › PNASNEXUS-PNASNEXUS-2023-00671R-s01.pdf]

Kinesin and Myosin Motors Compete to Drive Rich Multi-Phase Dynamics in Programmable Cytoskeletal Composites

Daisy H. Achiriloaie[1,2], Christopher J. Currie[1], Jonathan Michel[3], Mehrzad Sasanpour[1], Christopher Gunter[4], K. Alice Lindsay[5], Michael J. Rust[6], Janet Y. Sheung[2], Parag Katira[4], Moumita Das[3], Jennifer L. Ross[5], Ryan J. McGorty[1], Rae M. Robertson-Anderson[1,]*

[1]Department of Physics and Biophysics, University of San Diego, San Diego, California 92110, United States

[2]W. M. Keck Science Department, Scripps College, Pitzer College, and Claremont McKenna College, Claremont, California 91711, United States

[3]School of Physics and Astronomy, Rochester Institute of Technology, Rochester, New York 14623, United States

[4]Department of Mechanical Engineering, San Diego State University, San Diego, CA 92182, United States

[5]Department of Physics, Syracuse University, Syracuse, New York 13244, United States

[6]Department of Molecular Genetics and Cell Biology, University of Chicago, Chicago, Illinois 60637, United States

**randerson@sandiego.edu*

Supplementary Information

Contents:

SI Methods: Detailed descriptions of all methods and materials.

Table S1: Parameters used in mathematical model and simulations.

Table S2: Comparison of average speeds $\langle v \rangle$ and corresponding standard deviations σ measured with PIV and DDM.

Movie S1: Four sample time-series of actin-microtubule composites exhibiting *Slow* dynamics.

Movie S2: Four sample time-series of actin-microtubule composites exhibiting *Fast* dynamics.

Movie S3: Four sample time-series of actin-microtubule composites exhibiting *Multimode* dynamics.

Figure S1: Cartoon of phase space of different composite formulations.

Figure S2: Two-dimensional and azimuthally-averaged DDM image structure functions for 9 additional time-series with *Slow, Fast,* and *Multimode* dynamics.

Figure S3: Temporal color maps for 9 time-series with *Slow, Fast,* and *Multimode* dynamics.

Figure S4: PIV vector fields for 9 additional time-series with *Slow, Fast,* and *Multimode* dynamics.

Figure S5: Fits of 9 additional *Slow, Fast,* and *Multimode* speed distributions to Schulz functions.

Figure S6: Time course of average filament speeds and orientations for 9 additional time-series with *Slow, Fast,* and *Multimode* dynamics

Figure S7: Stacked 3-dimensional confidence ellipse plots show the relationships between average speed $\langle v \rangle$, anisotropy factor A_F, and skewness S_K for different composite formulations.

Figure S8: Sample plot showing simulation mechanics.

Figure S9: Simulation snapshots for three independent trials for each composite formulation.

Supplementary Methods:

Protein Preparation: Rabbit skeletal actin monomers (Cytoskeleton, AKL99, Lot#139), biotin-actin monomers (Cytoskeleton, AB07, Lot#49), porcine brain tubulin dimers (Cytoskeleton, T240, Lot#121), biotin-tubulin dimers (Cytoskeleton, T333P, Lot#27), rhodamine-labeled tubulin dimers (Cytoskeleton, TL590M, Lot#31), and myosin-II (Cytoskeleton, MY02, Lot#19), are reconstituted and flash-frozen into single-use aliquots according to previously described protocols (1).

Biotinylated kinesin-401 is expressed in Rosetta (DE3)pLysS competent *E. coli* (ThermoFisher) grown on selective media plates for 16-18 hours at 37°C. Fifteen colonies are added to a 5 ml starter culture of selective LB media and grown for 2 hours at 37°C/250rpm before adding to 400 ml of selective LB media. Cells are grown at 37°C/250rpm to OD 0.6-0.9 at 600 nm, then induced at 20°C/250rpm for 18 hours with 1mM Isopropyl β-D-1-thiogalactopyranoside (IPTG), and pelleted at 5,000 rpm for 10 minutes at 4°C before being frozen at -80°C for 1 hour. Cells are lysed in lysis binding buffer (50 mM PIPES, 4 mM $MgCl_2$, 20 mM imidazole, 10 mM β-mercaptoethanol, 50 μM ATP, one protease inhibitor tablet per 10 ml, 1.1mg/ml PMSF, 1.1mg/ml lysozyme) via sonication for 3 mins, pulsing every 20 seconds, then pelleted for 30 mins at 40,000 x g at 4°C, filtered through a 0.22 uM filter, and incubated with 1 ml nickel (Ni-NTA) agarose beads (Qiagen) for 2 hours on a rocker at 4°C. The lysate/bead mixture is passed through a chromatography column then washed with 15 ml buffer (50mM PIPES, 4mM MgCl2, 20mM imidazole, 10 mM β-mercaptoethanol, 50 μM ATP, one protease inhibitor tablet per 10ml) before 1 mL fractions are eluted in (50 mM PIPES, 4 mM MgCl2, 20 mM imidazole, 10mM β-mercaptoethanol, 50 μM ATP, one protease inhibitor tablet per 10 ml, 2 mM DTT, 0.05 mM ATP). An elution dot blot is performed to assess the most concentrated fraction which is run through a 40K MWCO desalting column for buffer exchange with PEM-100 with 0.1mM ATP, then mixed with 60% sucrose for a final concentration of 10% sucrose before being aliquoted and flash-frozen into single-use aliquots.

For composites that incorporate actin or microtubule crosslinking, actin-actin or microtubule-microtubule crosslinker complexes are prepared according to previously described protocols (2). In brief, biotin-actin or biotin-tubulin is combined with NeutrAvidin and free biotin at a ratio of 2:2:1 protein:free biotin:NeutrAvidin.

Immediately prior to experiments: myosin-II is purified as previously described (1) and stored at 4°C, and kinesin clusters are formed by incubating the dimers at a 2:1 ratio with NeutrAvidin (ThermoFisher) with 4 μM DTT for 30 minutes at 4°C.

Active Cytoskeleton Composite Preparation: Actin-microtubule composites are formed by polymerizing 2.32 μM unlabeled actin monomers and 3.48 μM tubulin dimers (5% rhodamine-labeled) in PEM-100 (100 mM PIPES, 2 mM $MgCl_2$, 2 mM EGTA) supplemented with 0.1% Tween, 10 mM ATP, 4 mM GTP, 5 μM Taxol, and 0.47 μM AlexaFluor488-phalloidin (Life Technologies, A12379) to label the actin.

For crosslinked composites, a portion of either the actin monomers or the tubulin dimers is replaced with equivalent crosslinker complexes to achieve the same overall actin and tubulin concentrations and crosslinker:protein ratios of R_A = 0.02 for actin or R_{MT} = 0.005 for microtubules. R_A and R_{MT} values are chosen to achieve similar lengths between crosslinkers d along actin filaments and microtubules ($d_A \simeq 60$ nm and $d_{MT} \simeq 67$ nm). As previously described (2), we estimate these values using $d_A = \frac{l_{monomer}}{2R}$, where $l_{monomer}$ is the length of an actin monomer, and $d_{MT} = \frac{l_{ring}}{26R}$, where l_{ring} is the length of a ring of 13 tubulin dimers. Crosslinking ratios are also tuned to be high enough to induce measurable changes in the viscoelastic properties compared to unlinked networks, but low enough to prevent filament bundling (2).

Actin and tubulin concentrations are chosen to be similar to those used in previous studies on myosin-driven actin-microtubule composites (1, 3, 4), and such that the mesh sizes for the actin and microtubule networks are comparable ($\zeta_A \simeq$0.96 μm and $\zeta_{MT} \simeq$1.44 μm, respectively), and the effective composite mesh size is

$\zeta_C \simeq (\zeta_A^3 + \zeta_{MT}^3)^{-1/3} \simeq 0.64$ µm (5). Further fine-tuning of the concentrations is achieved through a series of optimization experiments to identify a formulation space in which composites reliably form percolated networks and are visibly active on the timescale of minutes.

Composites are polymerized for 30 mins at 37°C, after which 1.86 μM unlabeled phalloidin is added and the composite is incubated for 10 mins at room temperature. 50 μM blebbistatin is added to inhibit myosin-actin interaction prior to de-activation via 488 nm illumination (1), and an oxygen scavenging system (45 μg/mL glucose, 0.005% β-mercaptoethanol, 43 μg/mL glucose oxidase, 7 μg/mL catalase) is added to reduce photobleaching. Finally, 0.47 μM myosin-II and 0.35 μM kinesin (pre-formed into complexes) are added. Concentrations of actin, tubulin, myosin-II and kinesin in composites are within reported physiological ranges of ~2.6 – 70 μM, ~1.3 – 19 μM, 0.4 – 4.8 μM, and 0.1 – 1.6 μM, respectively (6).

While myosin activity is controlled by blebbistatin de-activation, kinesin starts to act on microtubules immediately, so the start of the activity time of each experiment, $T_A = 0$, is set as the time kinesin is added. Each sample is gently flowed into a ~1 mm (x) × 24 mm (y) sample chamber composed of a silanized (7) coverslip and microscope slide fused together by a ~100 µm thick parafilm spacer and sealed with epoxy, creating an airtight chamber. We see no visible signs of sample drift from leaking or heating as our control systems (no motors) display no discernible bulk motion or restructuring. We do note that in cases in which motors induce directional motion, the motion is preferentially along the long 'y' axis of the chamber. We do not expect this preferred directionality to artificially bias any other structural or dynamical features of the composite.

Fluorescence Microscopy: Imaging of AlexaFluor488-labeled actin and rhodamine-labeled microtubules comprising composites is performed using a Nikon A1R laser scanning confocal microscope with a 60× 1.4 NA oil-immersion objective (Nikon), a 488 nm laser with 488/525 nm excitation/emission filters, and a 561 nm laser with 565/591 nm excitation/emission filters. 488 nm illumination also locally activates myosin-II ATPase activity by de-activating blebbistatin as previously described (1, 3, 4). Time-series (videos) of 256 × 256 square-pixel (213 µm × 213 µm) images are collected at 2.65 fps for 1000 frames ($t = 0 - 377$ s $\simeq 6.28$ mins). Acquisition of the first video for each sample starts 5 mins after the addition of kinesin motors ($T = 5$ min) in the middle of the ~100 µm thick sample chamber. Each successive video is collected in a different field of view of the same sample until there is no longer any discernible restructuring or motion ($T \simeq 45 - 120$ mins). 7-15 videos, each spanning acquisition times of $t = 0 - 377$ s, are collected for each of the six composite formulations (no crosslinking, actin crosslinking and microtubule crosslinking; with kinesin and with kinesin and myosin).

Differential Dynamic Microscopy (DDM): DDM is performed separately on the actin and microtubule channels of each 1000-frame video using custom written python scripts as described previously (1, 4). Image structure functions are determined by taking the square of 2D Fourier transforms of differences between an image at time t and one at $t + \Delta t$. This yields the instantaneous image structure function, $D_i(q_x, q_y, \Delta t, t_v)$ where q_x and q_y are x and y components of the wave vector $\vec{q}$. As typically done in DDM analysis, we average D_i over all times t (frames) of a given video, and all wave vectors $\vec{q}$ with the same magnitude q, to determine the 1D image structure function $D(q, \Delta t)$ that can be fit to various models. We fit $D(q, \Delta t)$ versus Δt for each wave vector q to the sum of either one or two Schulz speed distributions:

$$A\left(1 - \left(\left[f\left(\frac{\tau_1(Z_1+1)}{Z_1 * \Delta t} * \frac{\sin(Z_1 * \arctan(\theta_1))}{(1+\theta_1^2)^{\frac{Z_1}{2}}}\right)\right] + \left[(1-f)\left(\frac{\tau_2(Z_2+1)}{Z_2 * \Delta t} * \frac{\sin(Z_2 * \arctan(\theta_2))}{(1+\theta_2^2)^{\frac{Z_2}{2}}}\right)\right]\right)\right) + B,$$

where amplitude A, background B, decay times τ_1 and τ_2, amplitude fraction f, and Schulz numbers Z_1 and Z_2 are q-dependent free parameters, and $\theta_n = \frac{\Delta t}{\tau_n(Z_n+1)}$ (8). Schulz numbers characterize the speed distributions $P(v) = \frac{v^z}{Z!}\left(\frac{Z+1}{\bar{v}}\right)^{Z+1} \exp\left[-\frac{v(Z+1)}{\bar{v}}\right]$ where $Z = \left(\frac{\bar{v}}{\sigma}\right)^2 - 1$. We use the functional form of

$D(q,\Delta t)$ and the corresponding Schulz distribution fits to divide our data into three dynamical classes: *Slow*, *Fast* and *Multimode*. *Slow* data as those with $D(q,\Delta t)$ curves that exhibit a single flat decorrelation plateau and are well-fit to a single Schulz distribution (i.e., $f = 1$). *Fast* data are classified by $D(q,\Delta t)$ curves that are also well-fit to a single Schulz distribution, but have decorrelation plateaus that exhibit pronounced Δt-dependent oscillations. *Multimode* data have $D(q,\Delta t)$ curves that display two distinct plateaus and are best fit to the sum of two Schulz distributions with comparable f values. $\tau(q)$ curves for each composite and time T are extracted from the corresponding $D(q,\Delta t)$ fits.

For *Slow* and *Fast* data, in which one distribution describes the data, there are 4 free parameters (A, B, τ_1, Z_1). For *Multimode* data, this number increases to 7 (adding τ_2, Z_2, f). For each video these fits are performed over 40 different q values in the range $q = 0.8 - 2\ \mu\text{m}^{-1}$ (~3 – 8 μm), from which we extract $\tau(q)$ curves for the actin and microtubule channels of each of the 7-15 statistically different videos we collect for each of the six composite formulations.

Unreliable fits to the data would result in noisy $\tau(q)$ curves or curves that do not display power-law behavior over the entire q range. On the contrary, we find that all composites for all times T during activity exhibit $\tau(q) \sim q^{-1}$ scaling indicative of ballistic motion from which we compute the average speed $\langle v \rangle$ by fitting to $\tau(q) = (\langle v \rangle q)^{-1}$. We determine the error associated with the measured $\langle v \rangle$ using two methods. First, we compute $\langle v \rangle$ from each individual (τ,q) pair (i.e., $v = 1/\tau q$) and determine the standard error of the distribution of those values. Secondly, we use the Schulz parameter Z determined from our $D(q,\Delta t)$ fits and our measured $\langle v \rangle$ to compute the standard deviation σ and corresponding standard error via the relation $Z = \left(\frac{\bar{v}}{\sigma}\right)^2 - 1$. The error bar for each data point in Fig 3 represents the larger of the two standard error values.

All composites exhibit $\tau(q) \sim q^{-1}$ scaling indicative of ballistic motion (9) and the average speed $\langle v \rangle$ is computed by fitting $\tau(q)$ to $\tau(q) = (\langle v \rangle q)^{-1}$. Error bars shown in Fig 3 represent the standard error of the distribution of speeds computed from each individual q value (i.e., $v = 1/\tau q$) in the range over which we fit $D(q,\Delta t)$.

To determine the degree to which dynamics deviate from radial symmetry, implying directionality, we compute the anisotropy factor A_F of $D_i(q_x, q_y, \Delta t, t)$ in q-space by computing $A_F(q,\Delta t, t) = \int_0^{2\pi} D(q,\Delta t,\theta)\cos(2\theta)\, d\theta / \int_0^{2\pi} D(q,\Delta t,\theta) d\theta$ and averaging over q, Δt and t (10, 11). θ is defined relative to the positive y-axis such that $A_F > 0$ and $A_F < 0$ correspond to motion along the y- and x-direction, respectively, and $A_F = 0$ indicates isotropic motion.

To evaluate the time-dependence of dynamics over short timescales (within the time t of a single video), we also investigate the temporal distribution of instantaneous image structure functions $D_i(q_x, q_y, \Delta t, t)$ for a given q. For steady-state dynamics, one would expect this distribution to be Gaussian. Deviations from Gaussianity indicate sporadic events which cause larger than typical structural decorrelations. We quantify this non-Gaussian behavior by evaluating the skewness, $S_K = (\langle D_i - D \rangle)^3 / (\langle (D_i - D)^2 \rangle)^{3/2}$, where the average is over Δt and q.

Particle Image Velocimetry (PIV): PIV analysis is performed using the GPU-accelerated version of Open-PIV (12). We use interrogation windows of 8 × 8 square-pixels, with a 4 × 4 square-pixel overlap, to generate 64 × 64 grids of velocities for both the microtubule and actin channel of each time-series. Average velocities $\vec{v}$ for each interrogation window are determined from image pairs separated by Δt =10 frames (~3.77 s), with the starting frame for each successive interval overlapping with the ending frame for the previous interval. From the measured velocities, we determine the distribution of individual speeds $v(t)$, and velocity orientations $\theta(t)$ over the course of a video. Because of the heterogeneous spatial distribution of fluorescent material, the signal-to-noise ratio of estimated velocities varied appreciably. To identify and exclude spurious velocities during statistical analysis, we rejected those points for which the signal-to-noise

ratio was less than 2. To fit Schulz distributions for flow speed, we first partitioned velocities into bins of width 50 nm/s, computing the fraction of velocities in each bin. Schulz distribution parameters were then chosen by minimizing the mean square difference between the predicted statistical weight assigned to each bin for a given choice of parameters and the actual fraction of speeds in each bin. To visualize velocity fields using vector plots, we smoothed vector fields to eliminate spurious vectors in two steps. First, we removed vectors with unsatisfactory signal-to-noise ratios, and replaced the velocity vectors at the corresponding locations by local mean method, as implemented in the OpenPIV Spatial Analysis Toolbox. In the local mean approach, an invalid vector is iteratively replaced by the mean of all valid velocity vectors in a local patch centered about the site of the spurious vector. Here, we use a 3×3 averaging region. If at some location, no valid vectors are available at adjacent grid sites for a given iteration, a velocity field is not computed. The process is repeated until all spurious vectors are replaced. Next, we removed velocities that had a component that was more than 2 standard deviations greater than the global mean, and replaced them by the local mean method described above. Arrows plotted in Fig 4B and Fig S3 represent the local velocity on a regular Cartesian grid, with arrow length proportional to speed. Visualizations at different video times t are superposed, with arrow color representing t.

Spatial Image Autocorrelation (SIA): SIA analysis is performed on the actin and microtubule channels separately for each frame of each video using custom Python scripts, previously validated for similar active systems (4, 13, 14). SIA measures the correlation in intensity g_I of two pixels in an image (video frame) as a function of separation distance $\vec{r}$ (13). That is, $g_I(\vec{r}) = \langle I(\overrightarrow{r'} + \vec{r}) I(\overrightarrow{r'}) \rangle_{\overrightarrow{r'}}$ where $\overrightarrow{r'}$ represents the position of each pixel within an image. We perform an azimuthal average to generate $g_I(r)$ where r is the magnitude of $\vec{r}$. Computationally, this autocorrelation function is found by taking the Fourier transform of the image, multiplying by its complex conjugate, and applying an inverse Fourier transform. To normalize the autocorrelation functions so that the maximum is 1 (i.e., $g_I(0) = 1$), we subtract from each image the mean of that image and divide the image by its standard deviation before performing the Fourier transforms. From the resulting expression, $g_I(r) = \frac{F^{-1}(|F(I(r))|^2)}{[I(r)]^2}$, we radially average $F(I)$ to compute a single average correlation value for each lengthscale r of a given image, independent of direction. We use a spatial resolution of 1 pixel (832 nm) and perform SIA over the entire 256 × 256 square-pixel (213 μm)^2 image. We also perform SIA on skeletonized versions of the same images to reduce potential noise from introducing artifacts. The trends we observe in skeletonized and raw images are statistically indistinguishable. Finally, we perform this same SIA analysis on images from simulations (see Figs 6, S9). Correlation curves shown in (i) Fig 6F,G and (ii) Fig 6H,I, are averages across (i) 100 microscope images from 3 independent time-series and (ii) simulation snapshots from 3 independent runs (see below). Error bars indicate standard error.

Computational Model: To predict the restructuring of the composites due to motor activity, we develop a minimal model, based on the framework described in Ref 18 and references therewithin, that captures the key energetic components of the composites. We define the available space as a hexagonal grid with periodic boundary conditions. Each grid point can be occupied by an actin or microtubule filament center or can be empty. The filaments can interact with neighboring filaments within reach, via 1) motor-generated forces that can either pull the interacting filaments towards each other or push them away from each other; or 2) crosslinks that increase the friction forces on the interacting filaments. The movement of a filament center to a neighboring grid point within a small temporal time step is then a stochastic event whose probability can be calculated by the standard solution to the Fokker-Planck equation given by

$$p_{ij}(x \geq l) = 1 - \frac{1}{2}(1 + \text{erf}\left(\frac{l-\mu_{ij}}{\sigma_i\sqrt{2}}\right)) \qquad \dots (1),$$

where l is the distance to the next grid point j in a particular direction, μ_{ij} is the average advection induced displacement from the current grid location in the direction from i to j, and σ_i is the diffusion-based root mean squared (rms) 1D displacement of the filament along the direction to the specific grid point. The subscript i represents a specific filament in the model and j represents a neighboring grid point. The average advection-induced displacement along a given direction, μ, is a function of time elapsed since the filament moves to the new grid point, and is calculated as

$$\mu_{ij}(t) = \frac{(\sum_{j\neq i} \boldsymbol{F_{ij}})\cdot\boldsymbol{\hat{\eta}}}{\gamma_i}\Delta t + \mu_{ij}(t - \Delta t) \quad \dots (2),$$

where $\boldsymbol{F_{ij}}$ is the force from the motors between filament i and a same-type (actin or microtubule), interacting filament j, given by the force per motor (F_m or F_k for myosin or kinesin, respectively) times the number of motors per filament (N_m or N_k). The direction of $\boldsymbol{F_{ij}}$ is along the line joining the two filament centers and can be attractive or repulsive depending on filament orientations; $\boldsymbol{\hat{\eta}}$ is the unit vector along the direction of motion to the neighboring grid point; and Δt is the time-step for which the probability of motion is being calculated. γ_i is the effective friction factor, given by the sum of the viscous drag on filament i, (γ_A or γ_{MT} for actin or microtubule, respectively), the protein friction from all motors between interacting filaments of similar type ($\gamma_m * N_m * N_{A,i}$ or $\gamma_k * N_k * N_{MT,i}$) and the protein friction from crosslinks between similar type filaments ($\gamma_X * N_{A,i}$ or $\gamma_X * N_{MT,i}$). γ_m (γ_k) is the friction factor per myosin (kinesin) motor between two filaments, $N_{A,i}$ ($N_{MT,i}$) is the number of actin (microtubule) filaments interacting with the current actin (microtubule) filament, and γ_X is the crosslinker friction factor between each interacting filament of similar type.

The diffusion based rms displacement of a filament in a specific direction is calculated using

$$\sigma_i = \sqrt{2D(t_i + \Delta t)},$$

$$D = \frac{k_B \mathrm{T}}{\gamma_i} \quad \dots (3),$$

Where t_i is the time a filament has been in grid location i, k_B is the Boltzmann constant, and T is the temperature of the system.

The movement of a filament center to a neighboring grid point occupied by another filament center is restricted sterically and can be only accomplished if the two filaments exchange positions. Thus, in such a scenario, the cumulative movement probability of filament i to a neighboring grid point containing filament j's center is given by

$p_{ij,c} = p_{ij} \times p_{ji} = p_{ji,c}$ … (4), which is the same for the filament at grid point j exchanging its location with filament at i.

Within the same spirit, the movement of a filament from grid point i to a neighboring grid point j that is empty can by calculated as

$$p_{ij,c} = p_{ij} \times 1 \quad \dots (5).$$

We purposefully choose a minimal approach to capture the dynamics to shed light on the competing factors of motor activity and friction from crosslinkers. Our model assumes a single length for all filaments while in experiments actin and microtubules assume a distribution of lengths. We treat all filaments as rigid rods while actin in experiments is semiflexible with a persistence length of ~17 µm. Our simulations are in 2D while experimental composites span 3D. Our future work will build these additional features into our model.

Some important justifications and derivations that underlie our modeling approach include:

1. While crosslinkers are often thought of as springs connecting different filaments, individual crosslinker bonds are reversible and transiently switch between bound and unbound states. When a force is applied

on this system, either internally via molecular motors, or via an external force (such as tension or shear), crosslinkers can slip along the length of the filaments as they transiently bind and unbind. The rate at which the filaments slip past each other (or past crosslinks on neighboring filaments) gives an estimate of the viscosity of the system. This slip also results in plastic deformation or yielding in the materials. A simplified molecular theory of viscosity, based on breaking of elastic bonds, slippage, and bond reformation between neighboring elements of a macroscale system, such as a crosslinked polymer network, is well described by Ref 18. The model relates describes the slipping rate v as a function of the force F driving this slip by the equation $v = F/(Nfk_s\tau_{on})$, where F is the driving force, N is the number of crosslinkers, f is the duty ratio (fraction of time the bonds are bound), k_s is the elastic stiffness (spring constant) of the linker, and τ_{on} is the average bond lifetime. This simplified approximation provides a good estimate of viscous, irreversible, rearrangements that can occur in crosslinked polymer networks with reversible crosslinker binding. The term in the RHS denominator is a measure of the viscosity of the system or the viscous drag on individual filaments. We want to once again clarify that the elastic nature of crosslinks is not being ignored, just being coupled with the transient nature of the crosslinker bonds. If τ_{on} is really large, i.e. the crosslinker bonds are really strong, then v will be really small, so there will be almost no permanent slip or plastic deformation. In this limit, the network will have an elastic response to external force.

2. Under the simplified assumptions of the Ref 18 model description, when an external force is trying to slip two crosslinked filaments past each other, this force is instantaneously balanced by the elastic force developed in the crosslinkers connecting the two filaments $F = k_s x$, where $x = vt$ is the average displacement between the two filaments, v is the slip velocity, and t is the average elapsed time. If an individual crosslinker bond has a finite average lifetime of τ_{on}, then the maximum force that is resisted by each crosslinker bond is $F = k_s v\tau_{on}$. If N is the total number of crosslinkers, and f is the average time a crosslinker stays bound, then the force balance becomes $F = Nfk_s v\tau_{on}$. Under the simplified assumption that both f and τ_{on} are constant, the resistive force is then proportional to the sliding velocity and the rest of the terms on the RHS can be treated as an effective drag on the filaments. While this assumption is indeed simplistic, as f and τ_{on} will change based on the force applied, in the regime we are considering, where the external forces per crosslinker are smaller than the characteristic dissociation force of the crosslinker bond, this assumption holds. The application of this force-velocity relationship has been used effectively to describe filament sliding and force generation characteristics of actin-myosin and microtubule-kinesin systems, as reviewed in Ref 18 and references therewithin.

3. We estimate the drag coefficient of individual myosin on actin and kinesin on microtubules from the RHS of the force equation $F = Nfk_s v\tau_{on}$. N is the number of myosins or kinesins interacting between actin and microtubule filaments, which is 1 for the drag from individual motors, but is included in the calculation of the total effective drag (see explanation for equation 2). f is calculated as $k_{on}/(k_{on} + k_{off})$ where k_{on} and k_{off} are the binding and unbinding rates of molecular motors with their respective filaments. Using the rates given in Ref 15 for skeletal muscle, the motor protein stiffness of 4 pN/nm based on estimates from Ref 18, and using $k_{off}^{-1} = \tau_{on}$, we estimate the drag per myosin on actin as ~0.2 pN.ms/nm. The value given for the drag coefficient in Ref 15 for actin sliding due to myosin activity is 0.4 pN.ms/nm. Both values are much higher than the fluid drag on the filament, as reported in Ref 17. Also, since they only balance the active force generation by attached myosin motors and any external force on the filament with this drag force, the drag coefficient should include the passive effects of bound motors, which are a combination of molecular friction as described above and fluid drag. A value in the range of 0.2-0.4 pN.ms/nm for myosin drag on actin is in line with a 2 pN force from single myosin motors moving actin filaments at speeds of ~5-10 μm/s. Similarly, drag coefficients for kinesin motors are calculated from duty ratio and off rates given in Ref 18 giving a value ~6 pN.ms/nm, which can be compared to the 5 pN force generated per motor and ~0.8 μm/s microtubule sliding speeds.

We implement our model on a 155 µm x 155 µm 2D space with a hexagonal lattice, where the lattice spacing is 2.5 µm. Initially, each lattice point is either occupied with a microtubule filament center, an actin filament center or is left empty using probabilities matching the average volume fraction occupied by these elements. The movement of the filaments is simulated in each iteration by calculating the likelihood of each possible movement, $p_{ij,c}$ for all grid points i and j, where at least one of them contains a filament center, and randomly picking one of these movements to occur based on these probabilities. Since each movement occurs over a timescale of Δt, the effective time progression per movement can be approximated by $\frac{2\Delta t}{\sum_{i,j} p_{ij}}$ ($i \neq j$, at least i or j are occupied by a filament center) at each iteration step. We take the value of Δt as 0.1 s, at the start, such that both the rms displacement due to diffusion and the average advection distance due to motor driven forces are both smaller than the grid size, but dynamically adjust the Δt at the next iteration step to match the effective time progression from the previous iteration step. The simulation is run for $T_S = 10^6$ iterations, which we find is sufficient to reach quasi-steady state. Specifically, running the simulation for $0.5T_S$, $0.8T_S$, T_S, and $1.2T_S$ iterations, we observe insignificant change in the filament distributions for $\geq 0.8T_S$. The model calculations and simulations are coded in python and the scripts are available on GitHUB (https://github.com/compactmatterlab/active-filament-networks). A cartoon depiction of the model is shown in Fig S8 and numerical values for all model parameters are included in Table S1. We perform three independent simulation runs for each composite formulation for error analysis (see Fig S10).

To quantify the degree of clustering and segregation of the different filaments, we compute the probability distributions of like ($g_{A-A}(r)$, $g_{MT-MT}(r)$) and unlike ($g_{A-MT}(r)$, $g_{A-MT}(r)$) filaments a radial distance r from a given actin filament (A) or microtubule (MT) as:

$g_{A-A}(r) = <\frac{N_A(r)}{f_A N(r)}>$ and $g_{MT-MT}(r) = <\frac{N_{MT}(r)}{f_{MT} N(r)}>$ for like filaments, and

$g_{A-MT}(r) = <\frac{N_{MT}(r)}{f_{MT} N(r)}>$ and $g_{MT-A}(r) = <\frac{N_A(r)}{f_A N(r)}>$ for unlike filaments.

In the above, $N_{A/MT}(r)$ is the number of actin/microtubule neighbors at distance r from a specific filament, $f_{A/MT}$ is the volume fraction of actin/microtubules in the simulation space, and $N(r)$ is the maximum number of possible neighbors a distance r from the specific actin/microtubule filament. An increase in $g_{A/MT-A/MT}(r)$ above 1 indicates clustering of actin/microtubules, while a decrease in $g_{A/MT-MT/A}(r)$ below 1 indicates segregation of actin/microtubules from microtubules/actin. We perform correlation analysis up to $r = 15$ µm which we found sufficient to capture most of the correlation decay with r. Large radial distances display periodicity due to the periodic boundary conditions incorporated into the model. Spatial analysis algorithms also exclude filaments located at the maximum radial analysis distance or less from the simulation boundaries, to prevent the boundaries from skewing the results. Correlation analysis data shown in Fig 6 are averages over all filaments across three statistically independent replicates with error bars representing the standard error.

	Description	Value	Reference
**Total Grid Size**		150 µm x 150 µm	
**% actin filaments**	% of 2D space taken up by actin filament	40%	experimental
**% microtubules (MT)**	% of 2D space taken up by MTs	15%	experimental
**Grid spacing (*l*)**		2.5 µm	
**Filament length**	Length of each actin filaments and MT	5 µm	experimental
$\boldsymbol{F_m}$	Force generated per myosin motor	3 pN	(15)
$\boldsymbol{F_k}$	Force generated per kinesin motor	6 pN	(16)
$\boldsymbol{N_m}$	Number of myosin motors per actin-actin interaction	10	experimental
$\boldsymbol{N_k}$	Number of kinesin motors per actin-actin interaction	5	experimental
$\boldsymbol{\Delta t}$	Time increment	100 ms	
$\boldsymbol{\gamma_A}$	Viscous drag on an actin filament	0.005 pN·ms/nm	(17)
$\boldsymbol{\gamma_{MT}}$	Viscous drag on a microtubule filament	0.01 pN·ms/nm	(18)
$\boldsymbol{\gamma_m}$	Viscous drag on the filament due to single myosin motor binding	0.3 pN·ms/nm	(15)
$\boldsymbol{\gamma_k}$	Viscous drag on the filament due to single kinesin motor binding	6 pN·ms/nm	(18)
$\boldsymbol{\gamma_X}$	Viscous drag on the filament due to single cross-linker binding	10 pN·ms/nm	lower-bound (19)
**T**	Temperature of the system	290 K	

Table S1: Parameters used in mathematical model and simulations. Specific numerical values of parameters are chosen to match experimental conditions, including the concentrations of actin, microtubules, motors and crosslinkers. Values for motor forces and viscous drag terms are based on literature values as specified in the table.

Class	Channel	Method			
		PIV		DDM	
		<v>	s	<v>	s
Slow	microtubule	0.48	0.33	0.27	0.07
	actin	0.23	0.17	0.33	0.09
Fast	microtubule	1.49	0.18	1.91	0.24
	actin	1.60	0.21	1.77	0.23
Multimode	microtubule - v1	0.75	0.23	0.82	0.19
	actin - v1	0.80	0.16	0.80	0.18
	microtubule - v2	0.19	0.12	0.17	0.01
	actin - v2	0.30	0.19	0.18	0.01

Table S2. Comparison of average speeds $\langle v \rangle$ and corresponding standard deviations σ measured with PIV and DDM. Average speed $\langle v \rangle$ and corresponding standard deviation σ for actin and microtubule channels of the videos shown in part A of Movies S1-S3, measured by fitting: (left) PIV speed distributions to one (purple, orange) or two (magenta) Schulz distributions, or (right) DDM $D(q, \Delta t)$ curves to functions that use one (purple, orange) or two (magenta) Schulz speed distributions. Note that all speeds are statistically indistinguishable between the two measurement techniques.

Movies:

Movie S1: Sample time-series of active actin-microtubule composite exhibiting *Slow* dynamics. (A) The example time-series used to demonstrate *Slow* dynamics of actin filaments (green) and microtubules (red) in Figs 2A, 3 and 4A, and (**B-D**) three additional time-series showing *Slow* dynamics.

Movie S2: Sample time-series of active actin-microtubule composite exhibiting *Fast* dynamics. (A) The example time-series used to demonstrate *Fast* dynamics of actin filaments (green) and microtubules (red) in Figs 2A, 3 and 4A, and (**B-D**) three additional time-series showing *Fast* dynamics.

Movie S3: Sample time-series of active actin-microtubule composite exhibiting *Multimode* dynamics. (A) The example time-series used to demonstrate *Multimode* dynamics of actin filaments (green) and microtubules (red) in Figs 2A, 3 and 4A, and (**B-D**) three additional time-series showing *Multimode* dynamics.

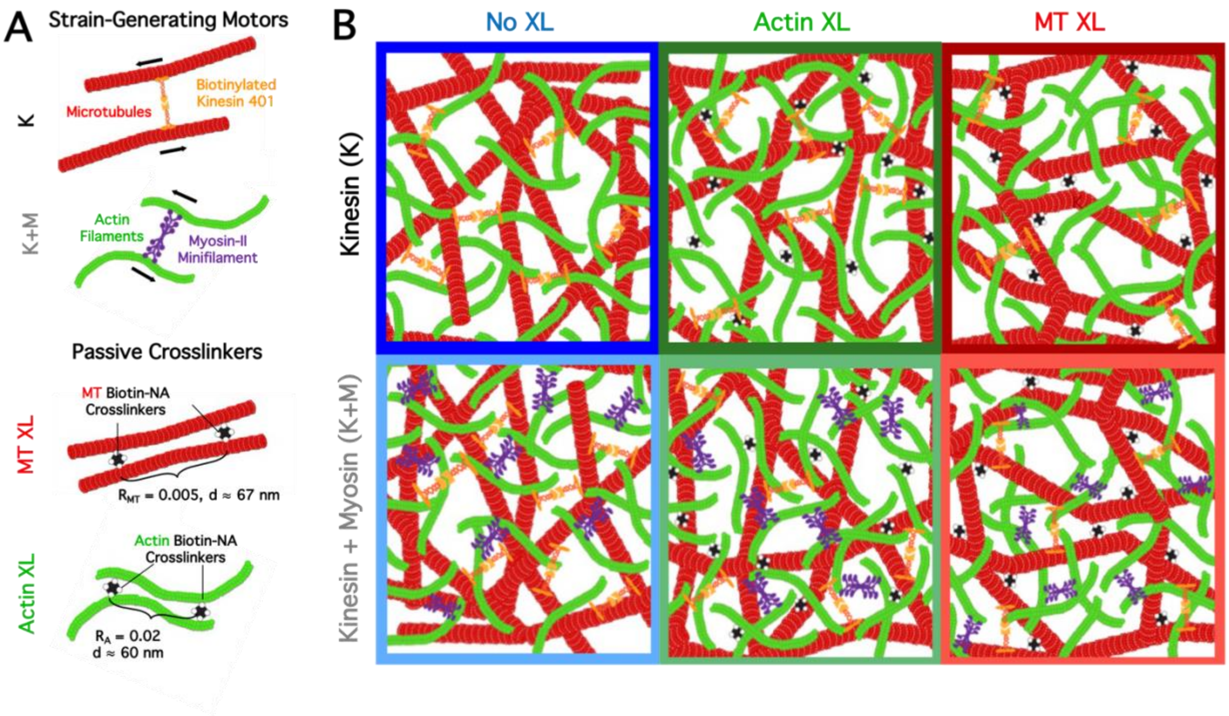

Figure S1: Cartoon of phase space of different composite formulations. (A) We co-polymerize actin monomers (2.32 µM) and tubulin dimers (3.48 µM) to form co-entangled composite networks of actin filaments (green) and microtubules (red). Static crosslinking is achieved using NeutrAvidin to link biotinylated actin filaments (Actin XL) or microtubules (MT XL). The crosslinker to protein molar ratio R is fixed at R = 0.02 for actin and R = 0.005 for microtubules to achieve similar distances d between crosslinks along the filaments. We incorporate kinesin clusters (orange) and myosin-II minifilaments (purple) as strain-generating motors to drive the composites out of steady-state. **B.** Cartoon of composite formulation space. We incorporate 0.35 µM kinesin (K) into composites with no static crosslinkers (No XL, dark blue box), actin-actin crosslinks (Actin XL, dark green box) and microtubule-microtubule crosslinks (MT XL, dark red box). For each kinesin-driven composite, we also examine the effect of adding 0.47 µM myosin (K+M) into composites with no static crosslinkers (No XL, light blue box), actin-actin crosslinks (Actin XL, light green box) and microtubule-microtubule crosslinks (MT XL, light red box).

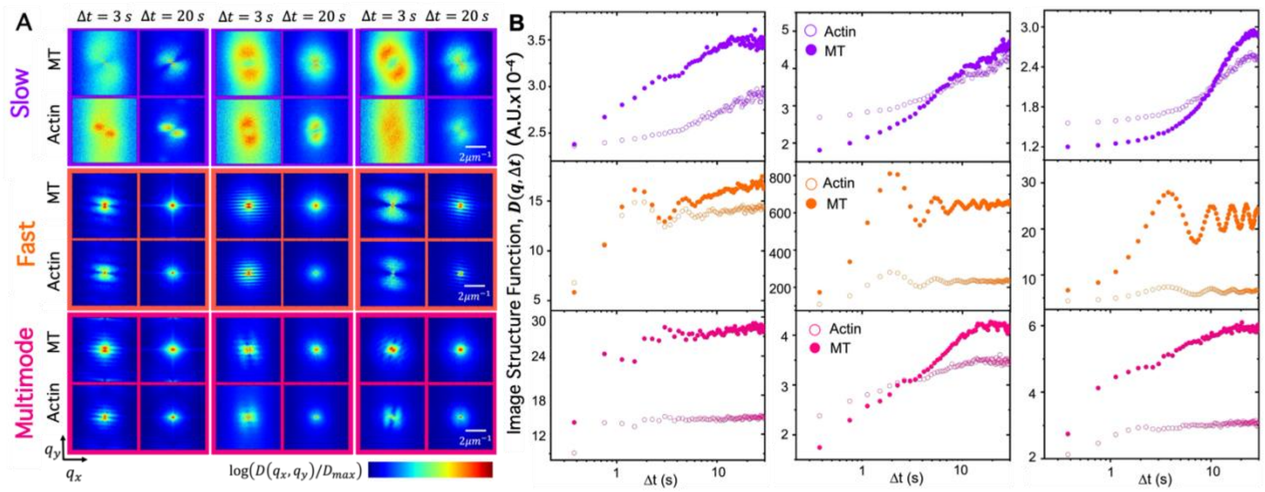

Figure S2: Two-dimensional and azimuthally-averaged DDM image structure functions for 9 additional time-series with *Slow*, *Fast*, and *Multimode* behavior. A. Two-dimensional image structure functions $D(q_x, q_y, \Delta t)$ computed for $\Delta t = 3$ s and $\Delta t = 20$ s for three representative time-series that display *Slow* (top rows, purple), *Fast* (middle rows, orange), and *Multimode* (bottom rows, magenta) characteristics. Colorscale is normalized separately for each image, and indicates the normalized value of each image structure function $[D(q_x, q_y, \Delta t)/D_{max}]$, with low (blue) and high (red) values indicative of lower or higher correlations. **B.** Azimuthally-averaged image structure functions $D(q, \Delta t)$ versus lag time Δt computed from $D(q_x, q_y, \Delta t)$ functions shown in (A) for microtubule (closed symbols) and actin (open symbols) channels evaluated at $q = 1.33\ \mu\text{m}^{-1}$.

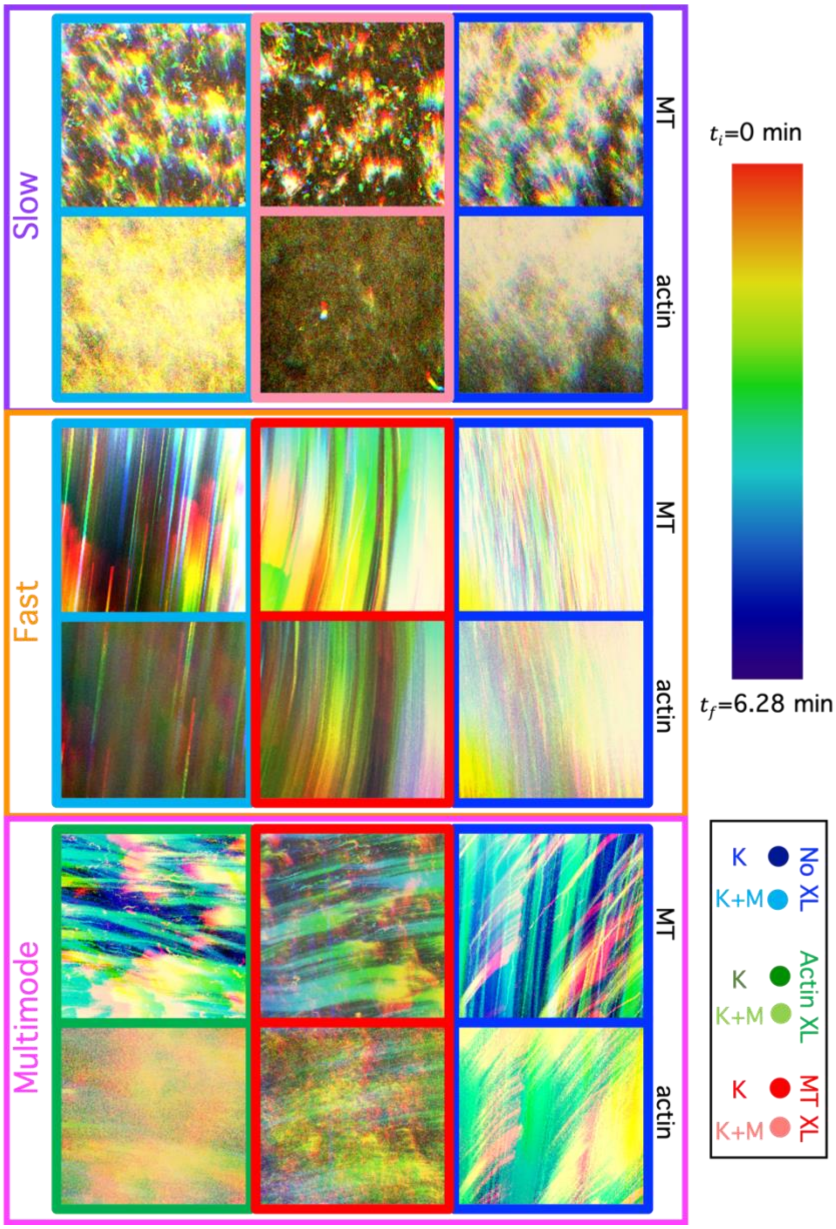

Figure S3: Temporal color maps for 9 additional time-series with *Slow*, *Fast*, and *Multimode* dynamics. Temporal color maps generated from the microtubule (top rows) and actin (bottom rows) channels of the nine different time-series analyzed in Fig S2, divided into *Slow* (top), *Fast* (middle), and *Multimode* (bottom) classes based on distinct $D(q, \Delta t)$ features shown in Fig 2A. Temporal color maps which colorize the features in each frame according to the time t the frame is captured during the video, as indicated by the colorscale ($t_i = 0$ min (red) to $t_f = 6.28$ min (purple)), depict the motion of the composites. The color outlining each map denotes the composite formulation according to the legend. Each 256 × 256 square-pixel image is 213 μm × 213 μm. The videos from which maps are generated are parts B-D of Movies S1-S3.

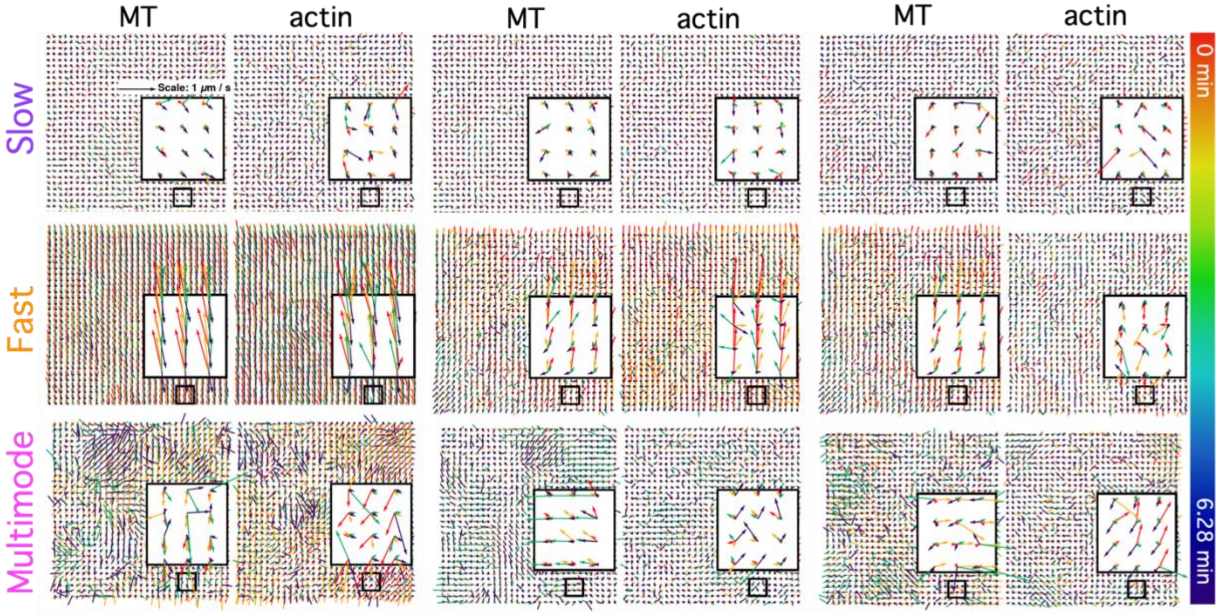

Figure S4: PIV vector fields for 9 additional time-series with *Slow*, *Fast*, and *Multimode* dynamics. PIV velocity vector fields for the microtubule (left) and actin (right) channels of parts B-D of Movies S1-S3 display *Slow* (top, purple, Movie S1), *Fast* (middle, orange, Movie S2), and *Multimode* (bottom, magenta, Movie S3) characteristics. Each arrow represents the average velocity vector for an 8×8 square-pixel region for $t = 0$ s (red), 125 s (yellow), 251 s (green) and 377 s (purple) as shown by the time-color scale. All vector fields are 213 μm × 213 μm and insets are zoom-ins of 25 μm × 25 μm square regions as indicated in the top-left field.

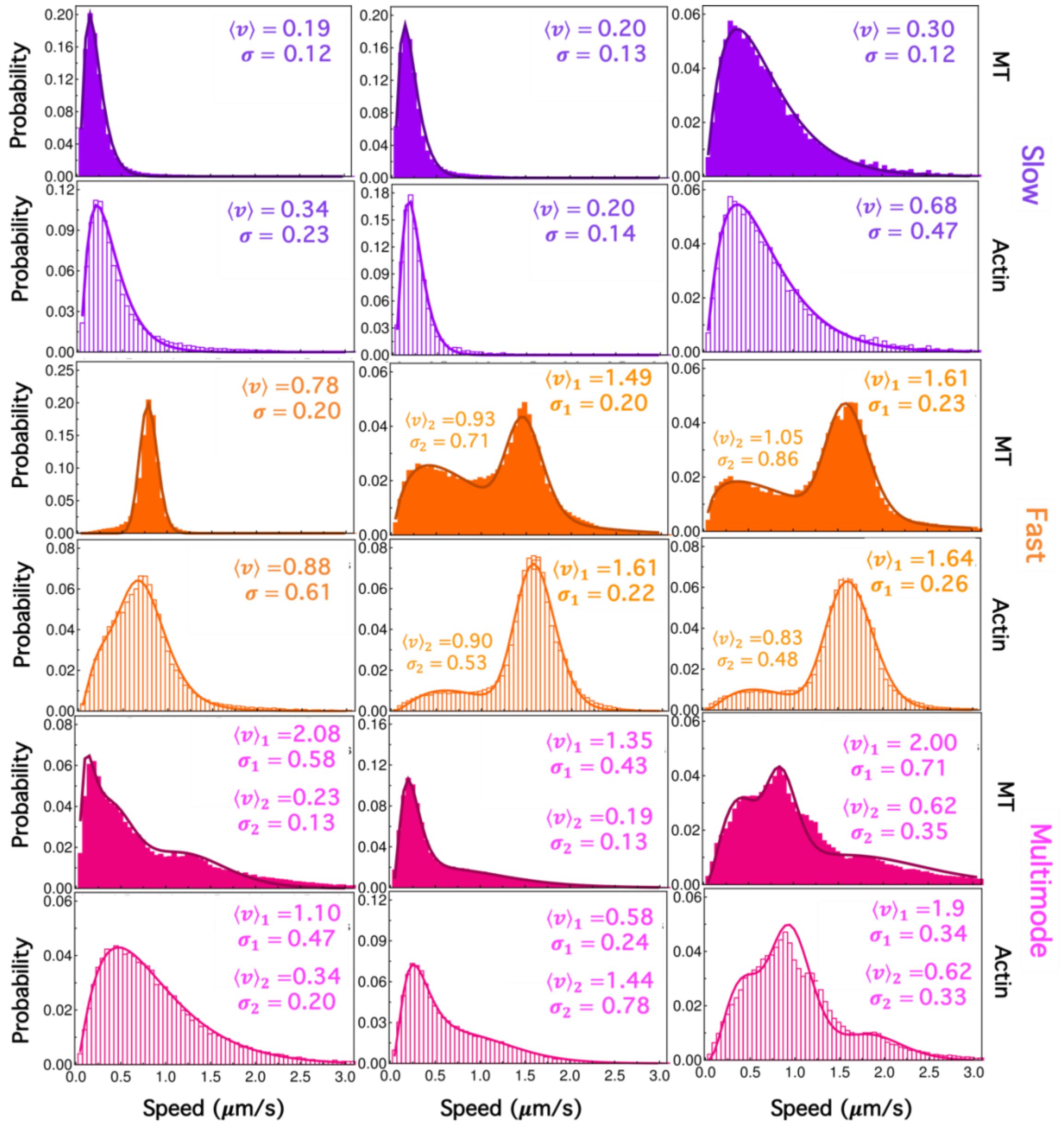

Figure S5: Fits of 9 additional *Slow*, *Fast*, and *Multimode* speed distributions to Schulz functions. Probability distributions of speeds determined from PIV for microtubules (filled) and actin (open) for the 9 *Slow* (top), *Fast* (middle) and *Multimode* (bottom) time-series analyzed in Fig S4 (B-D of Movies S1-S3). Dashed lines are fits to one or two Schulz distributions: $P(v) = \frac{v^z}{Z!}\left(\frac{Z+1}{\bar{v}}\right)^{Z+1} \exp\left[-\frac{v(Z+1)}{\bar{v}}\right]$ where $Z = \left(\frac{\bar{v}}{\sigma}\right)^2 - 1$ and $\bar{v}$ and σ are the average and standard deviation of the speed distribution. $\bar{v}$ and σ determined from each fit are listed in units of μm/s. *Multimode* distributions are best fit to a sum of two distributions with different $\bar{v}$ and σ values (denoted by subscripts 1 and 2). Some *Fast* distributions are also better fit to a sum of two Schulz distributions, but the second distribution is weighted significantly less than the first distribution.

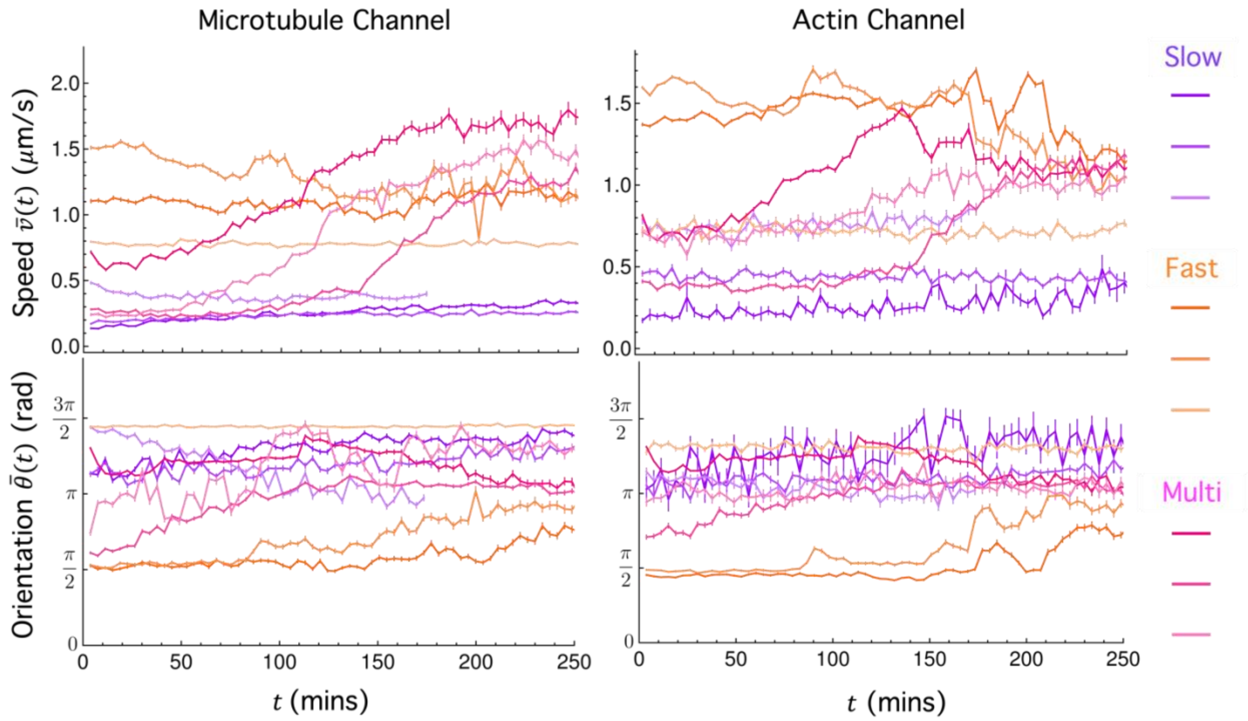

Figure S6: Short-time course of average filament speeds and orientations for 9 additional time-series with *Slow*, *Fast*, and *Multimode* dynamics. (Top) Average speed $\bar{v}(t)$ versus time t measured via PIV for the MT (left) and actin (right) channels of the 9 representative *Slow* (purple), *Fast* (orange) and *Multimode* (magenta) videos analyzed in Fig S4 (B-D of Movies S1-S3). $\bar{v}(t)$ for each time t is an average over all vector magnitudes in the PIV flow field associated with time t. **(Bottom)** Average velocity orientations $\bar{\theta}(t)$ versus t computed from the same vector fields following the same method as for $\bar{v}(t)$.

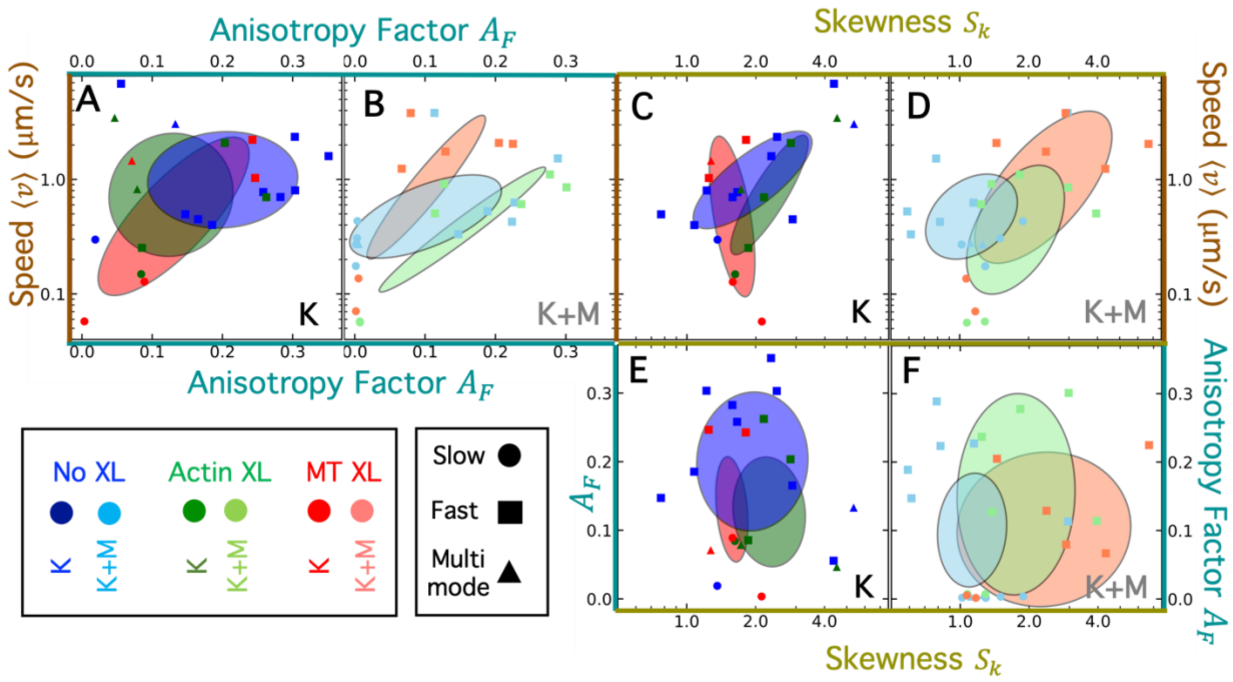

Figure S7: Stacked 3-dimensional confidence ellipse plots show the relationships between average speed $\langle v \rangle$ (brown axes), anisotropy factor $|A_F|$ (teal axes), and skewness S_K (gold axes) for different composite formulations. Data points correspond to the 106 data points plotted in Fig 5, with colors and symbols indicating the composite formulation and dynamic class, respectively, according to the legends. The ellipses enclose one standard deviation around the mean. Panels with darker shaded (A,C,E) and lighter shaded (B,D,F) ellipses display data for composites with kinesin (K) and both kinesin and myosin (K+M), respectively.

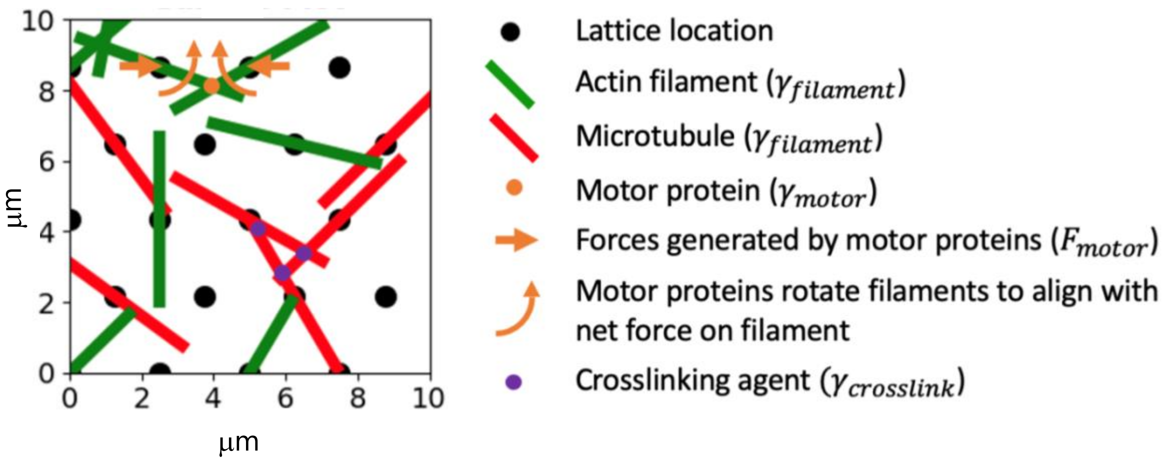

Figure S8: Sample plot showing simulation mechanics. The actin filaments and microtubules exist on a lattice of grid points. There is a drag ($\gamma_{filament}$) associated with their movement. Motor proteins exert forces which drive movement of the filaments (F_{motor}) but also exert drag (γ_{motor}). Crosslinking agents inhibit movement by exerting drag on their respective filaments ($\gamma_{crosslink}$).

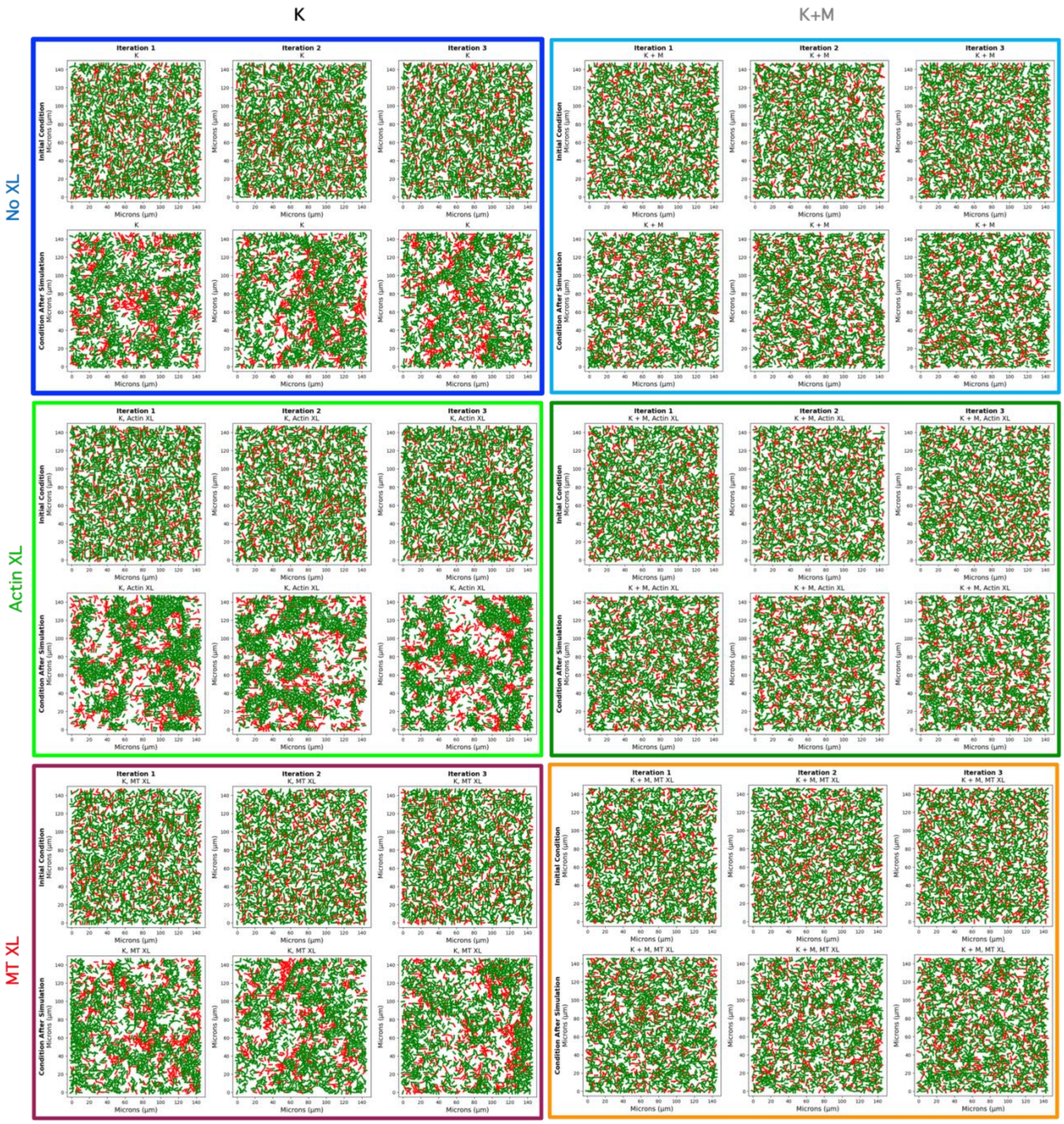

Figure S9: Simulation snapshots for three independent trials for each composite formulation. For each of the six composite formulations we investigate (indicated by the color-coded borders and labels) we simulate three independent iterations of the model. Correlation analysis data for each formulation (see SI Methods and Fig 6) are the corresponding average and standard error across the three trials. Color-coded borders enclose each formulation with lighter (right) and darker (left) shades denoting composites with and without myosin, respectively. For each formulation, the 6 images correspond to the initial (top row) and final (bottom row) states of the three independent runs (columns 1-3). All images show actin (green) and microtubules (red) comprising a 150 µm x 150 µm grid.

Supplemental Information References

1. G. Lee, *et al.*, Myosin-driven actin-microtubule networks exhibit self-organized contractile dynamics. *Sci. Adv.* **7**, eabe4334.
2. S. N. Ricketts, *et al.*, Varying crosslinking motifs drive the mesoscale mechanics of actin-microtubule composites. *Sci. Rep.* **9**, 12831 (2019).
3. J. Sheung, *et al.*, Motor-Driven Restructuring of Cytoskeleton Composites Leads to Tunable Time-Varying Elasticity. *ACS Macro Lett.* (2021).
4. G. Lee, *et al.*, Active cytoskeletal composites display emergent tunable contractility and restructuring. *Soft Matter* **17**, 10765–10776 (2021).
5. M. Doi, S. Edwards, *The Theory of Polymer Dynamics* (Clarendon Press, 1986).
6. D. N. Itzhak, S. Tyanova, J. Cox, G. H. Borner, Global, quantitative and dynamic mapping of protein subcellular localization. *eLife* **5**, e16950 (2016).
7. B. Edozie, *et al.*, Self-Organization of Spindle-Like Microtubule Structures. *Soft Matter* **15** (2019).
8. L. G. Wilson, *et al.*, Differential Dynamic Microscopy of Bacterial Motility. *Phys. Rev. Lett.* **106**, 018101 (2011).
9. R. Cerbino, V. Trappe, Differential Dynamic Microscopy: Probing Wave Vector Dependent Dynamics with a Microscope. *Phys. Rev. Lett.* **100**, 188102 (2008).
10. Z. Varga, J. W. Swan, Large scale anisotropies in sheared colloidal gels. *J. Rheol.* **62**, 405–418 (2018).
11. L. M. Walker, N. J. Wagner, SANS Analysis of the Molecular Order in Poly(γ-benzyl l-glutamate)/Deuterated Dimethylformamide (PBLG/d-DMF) under Shear and during Relaxation. *Macromolecules* **29**, 2298–2301 (1996).
12. A. Liberzon, *et al.*, *OpenPIV/openpiv-python: OpenPIV - Python (v0.22.2) with a new extended search PIV grid option* (Zenodo, 2020) https:/doi.org/10.5281/zenodo.3930343 (June 29, 2022).
13. C. Robertson, S. C. George, Theory and practical recommendations for autocorrelation-based image correlation spectroscopy. *J. Biomed. Opt.* **17**, 080801–080801 (2012).
14. R. McGorty, *rmcgorty/Image-Correlation* (2020) (February 21, 2021).
15. S. Stam, J. Alberts, M. L. Gardel, E. Munro, Isoforms Confer Characteristic Force Generation and Mechanosensation by Myosin II Filaments. *Biophys. J.* **108**, 1997–2006 (2015).
16. E. Meyhöfer, J. Howard, The force generated by a single kinesin molecule against an elastic load. *Proc. Natl. Acad. Sci. U. S. A.* **92**, 574–578 (1995).
17. E. Vazquez-Hidalgo, C. M. Farris, A. C. Rowat, P. Katira, Chemo-Mechanical Factors That Limit Cellular Force Generation. *Front. Phys.* **10** (2022).
18. J. Howard, *Mechanics of motor proteins and the cytoskeleton* (Sinauer Associates, Publishers, 2001).
19. F. Pincet, J. Husson, The Solution to the Streptavidin-Biotin Paradox: The Influence of History on the Strength of Single Molecular Bonds. *Biophys. J.* **89**, 4374–4381 (2005).
