# Supplementary figures and images for: Kinesin and myosin motors compete to drive rich multiphase dynamics in programmable cytoskeletal composites
Source: PNAS Nexus. 2023 Jul 31;2(8):pgad245. doi: 10.1093/pnasnexus/pgad245 (PMC10416814; doi:10.1093/pnasnexus/pgad245)

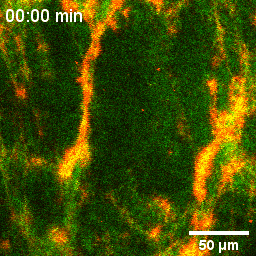

Supplement: pgad245_Supplementary_Data [file pgad245_supplementary_data.zip › PNASNEXUS-PNASNEXUS-2023-00671R-s11.gif]

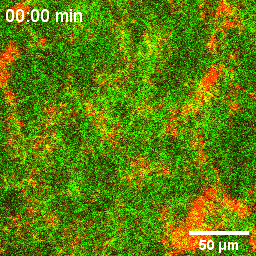

Supplement: pgad245_Supplementary_Data [file pgad245_supplementary_data.zip › PNASNEXUS-PNASNEXUS-2023-00671R-s12.gif]

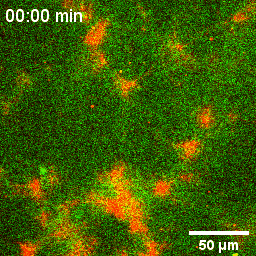

Supplement: pgad245_Supplementary_Data [file pgad245_supplementary_data.zip › PNASNEXUS-PNASNEXUS-2023-00671R-s13.gif]

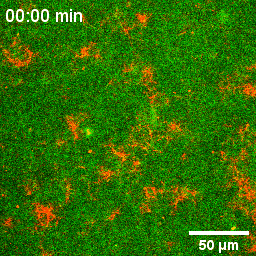

Supplement: pgad245_Supplementary_Data [file pgad245_supplementary_data.zip › PNASNEXUS-PNASNEXUS-2023-00671R-s03.gif]

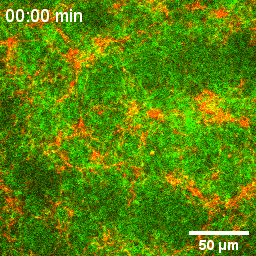

Supplement: pgad245_Supplementary_Data [file pgad245_supplementary_data.zip › PNASNEXUS-PNASNEXUS-2023-00671R-s04.gif]

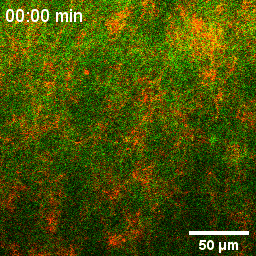

Supplement: pgad245_Supplementary_Data [file pgad245_supplementary_data.zip › PNASNEXUS-PNASNEXUS-2023-00671R-s05.gif]

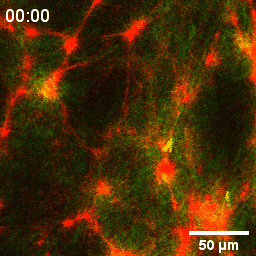

Supplement: pgad245_Supplementary_Data [file pgad245_supplementary_data.zip › PNASNEXUS-PNASNEXUS-2023-00671R-s06.gif]

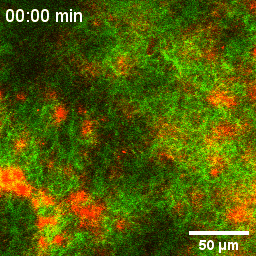

Supplement: pgad245_Supplementary_Data [file pgad245_supplementary_data.zip › PNASNEXUS-PNASNEXUS-2023-00671R-s07.gif]

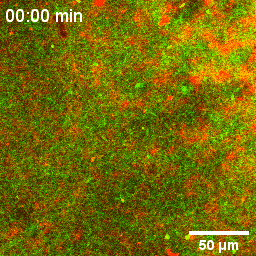

Supplement: pgad245_Supplementary_Data [file pgad245_supplementary_data.zip › PNASNEXUS-PNASNEXUS-2023-00671R-s08.gif]

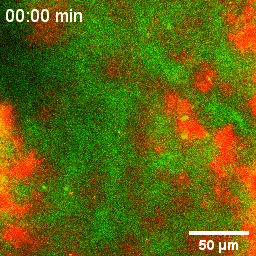

Supplement: pgad245_Supplementary_Data [file pgad245_supplementary_data.zip › PNASNEXUS-PNASNEXUS-2023-00671R-s09.gif]
